# A new elpistostegalian from the Late Devonian of the Canadian Arctic

**DOI:** 10.1038/s41586-022-04990-w

**Published:** 2022-07-20

**Authors:** Thomas A. Stewart, Justin B. Lemberg, Ailis Daly, Edward B. Daeschler, Neil H. Shubin

**Affiliations:** 1 Department of Organismal Biology and Anatomy, University of Chicago, Chicago, IL USA; 2 Department of Vertebrate Zoology, Academy of Natural Sciences of Drexel University, Philadelphia, PA USA; 3 Present Address: Department of Biology, The Pennsylvania State University, State College, PA USA

**Keywords:** Palaeontology, Taxonomy, Ichthyology

## Abstract

A fundamental gap in the study of the origin of limbed vertebrates lies in understanding the morphological and functional diversity of their closest relatives. Whereas analyses of the elpistostegalians *Panderichthys rhombolepis*, *Tiktaalik roseae* and *Elpistostege watsoni* have revealed a sequence of changes in locomotor, feeding and respiratory structures during the transition^1–9^, an isolated bone, a putative humerus, has controversially hinted at a wider range in form and function than now recognized^10–14^. Here we report the discovery of a new elpistostegalian from the Late Devonian period of the Canadian Arctic that shows surprising disparity in the group. The specimen includes partial upper and lower jaws, pharyngeal elements, a pectoral fin and scalation. This new genus is phylogenetically proximate to *T. roseae* and *E. watsoni* but evinces notable differences from both taxa and, indeed, other described tetrapodomorphs. Lacking processes, joint orientations and muscle scars indicative of appendage-based support on a hard substrate^13^, its pectoral fin shows specializations for swimming that are unlike those known from other sarcopterygians. This unexpected morphological and functional diversity represents a previously hidden ecological expansion, a secondary return to open water, near the origin of limbed vertebrates.

## Abstract (web summary)

A new elpistostegalian from the Late Devonian period has been discovered that shows disparity in the group and represents a previously hidden ecological expansion, a secondary return to open water, near the origin of limbed vertebrates.

## Main

Study of tetrapodomorph skulls, fins, axial skeleton and scalation has revealed the ways that feeding, respiration and appendage-based locomotion changed as fish shifted from aquatic to terrestrial lifestyles^15,16^. *P. rhombolepis*^1–3^, *T. roseae*^4–8^ and *E. watsoni*^9^ hold a special place in these analyses, showing a combination of plesiomorphic and apomorphic features that give insight into a sequence of anatomical changes in the origin of limbed taxa (that is, those in possession of digited appendages and lacking dermal rays). Now missing, however, is an understanding of the morphological, functional and ontogenetic diversity of the finned tetrapodomorphs most closely related to limbed forms. This is unfortunate, as isolated or fragmental specimens have controversially hinted at a wider range of diversity than is observed in more complete material^10–14^.

Here we describe a new finned tetrapodomorph that is closely related to *T. roseae* and *E. watsoni*. The new form shows an unexpected combination of characters, one that suggests a broad range in disparity among the closest finned relatives of limbed forms. The specimen was collected 1.5 km east of the site that yielded *T. roseae*, but from a slightly lower horizon in the Fram Formation of southern Ellesmere Island, Nunavut, Canada. We describe this new taxon and present a phylogenetic analysis to reveal its implications for understanding the evolution of the nearest relatives of limbed tetrapodomorphs. Comparison of the new taxon to other Frasnian-age forms allows a reinterpretation of isolated elements of previously uncertain affinity, thus, indicating a more widespread and diverse assemblage of tetrapod relatives than previously recognized.

## Geological framework

**Fig. 1:**
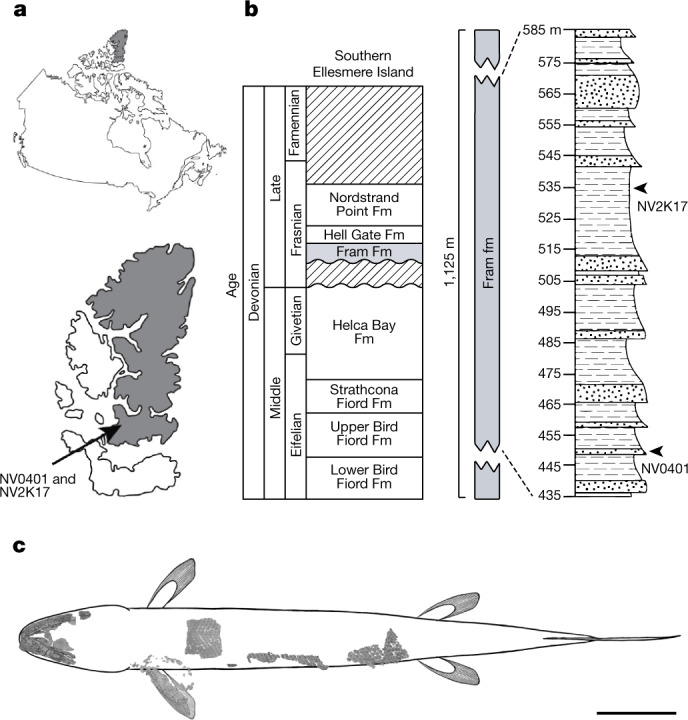
Locality and holotype of *Qikiqtania wakei* gen. et sp. nov. **a**, Specimen NUFV 137 was discovered on southern Ellesmere Island, Nunavut, Canada. **b**, The site, NV0401, lies 80 m below NV2K17, the site where *T. roseae* was discovered. **c**, Materials were µCT scanned and are shown here in dorsal aspect. General body shape based on specimen MHNM 06-2067 of *E. watsoni*^9^. Scale bar, 10 cm. Abbreviations: Fm, formation.

**Extended Data Fig. 1:**
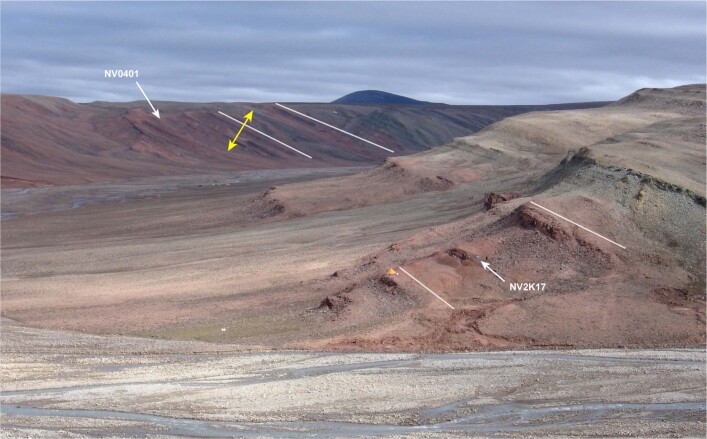
Photograph of the locality NV0401. Photograph showing the localities NV0401, where NUFV 137 was collected, and NV2K17, where *T. roseae* was collected. White arrows indicate sites of collection. Yellow arrows highlight approximate stratigraphic separation between the two horizons. White lines trace two additional horizons across the valley. A yellow tent, approximately 2.5 m across, is in the midground.

Embry and Klovan^17^ described the type section of the Fram Formation from a drainage feeding the eastern arm of Bird Fiord on southern Ellesmere Island. They indicate an early to middle Frasnian age for the Fram Formation on the basis of palynological spot samples, which were collected from near the base, the middle and top of the formation^17^. The Nunavut Paleontological Expeditions collected vertebrate remains from 2000 to 2008 at 16 sites from the Fram Formation within the type section. The holotype of *T. roseae* (NUFV 108), as well as all other *T. roseae* specimens, were collected from site NV2K17, which occurs in silty overbank floodplain deposits^18^ at 533 m above the base of the measured type section of Embry and Klovan^17^. The specimen discussed here (NUFV 137) was collected at site NV0401 (77° 10.235′ N, 86° 11.279′ W) from lower in the same section and 1.5 km from NV2K17 (Fig. 1a,b and Extended Data Fig. 1). Site NV0401 is about 453 m above the base of the type section and occurs in a medium-grained sandstone. The surface-collected NUFV 137 is the only specimen found at the site. NUFV 137 is older than *T. roseae* and was collected from a different facies in the floodplain deposits of the Fram Formation.

## Systematic palaeontology

*Sarcopterygii*^19^ Romer, 1955

Tetrapodomorpha^20^ Ahlberg, 1991

Elpistostegalia^21^ Camp and Allison, 1961

***Qikiqtania wakei*** gen. et sp. nov.

**Locality**. Canada, Nunavut, southern Ellesmere Island, near the eastern arm of Bird Fiord, Nunavut Paleontological Expedition site NV0401, 77° 10.235′ N, 86° 11.279′ W.

**Geological setting**. Fram Formation (Upper Devonian, early Frasnian Stage).

**Etymology**. *Qikiqtania* (pronounced ‘kick-kiq-tani-ahh’) is derived from Inuktitut word Qikiqtaaluk/Qikiqtani, the traditional name for the region where the fossil site occurs. The species designation is in memory of David Wake, an eminent evolutionary biologist and transformative mentor, late of the University of California at Berkeley.

**Holotype**. Nunavut Fossil Vertebrate Collection (NUFV) 137.

**Extended Data Fig. 2:**
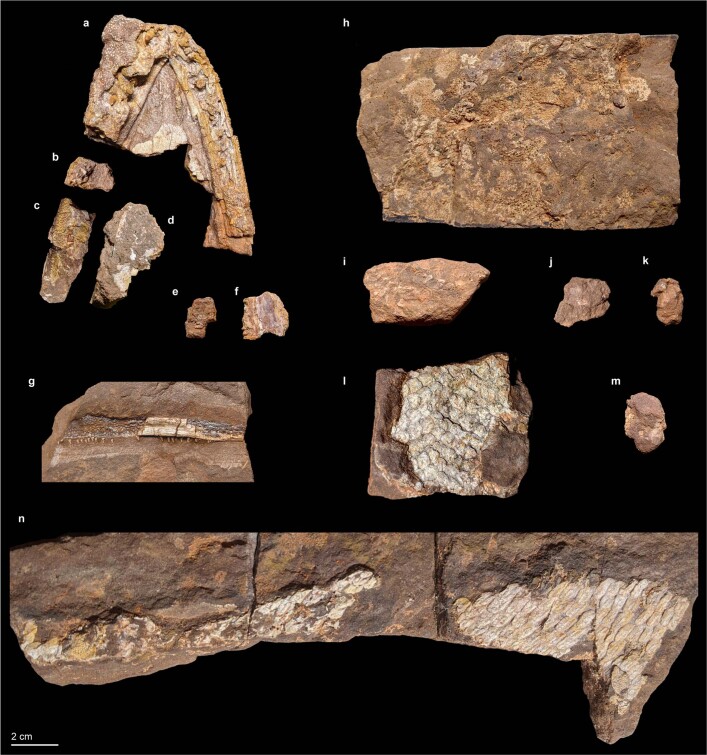
Photographs of NUFV 137. (a–f) Elements associated with the feeding apparatus. Elements in a–d are shown in Fig. 2 and Video S2. Element e is identified as parts of the palate and lower jaw due to the presence of multiple rows of both dorsally and ventrally facing teeth. The ventrally facing teeth are determined to be palatal in nature due to the expanded medial shagreen of denticles (likely part of the entopterygoid) that are bordered laterally by two uniform rows of larger teeth (likely the ectopterygoid and maxilla). This piece could not be definitively positioned relative to the other jaw elements due to absence of the corresponding broken tooth bases on the main lower jaw block. Element f is identified as part of a lower jaw on the basis of its curvature and dentition. (g) Maxilla. (h) Left pectoral fin, which is embedded in matrix, with exposed associated scales. (i) Fragment containing scales and lepidotrichia from a paired fin. (j, k) Fragments with undiagnosed vascularized endoskeletal elements. (l) Scale field from the dorsal midline, anterior is up. (m) Fragment containing scales from the lateral line series and flank. (n) Trunk scale field, anterior is left.

**Material***.* The description is based on a specimen from the NV0401 site that preserves the lower jaws, partial left upper jaw and palate in articulation, gulars, ceratohyals, an articulated left pectoral fin and articulated scales from the dorsal midline, flank and lateral line series (Fig. 1c, Extended Data Fig. 2 and Supplementary Video 1). The jaw material was physically prepared at the Academy of Natural Sciences of Drexel University. Computed tomography scans were collected at The University of Chicago’s PaleoCT scanning facility (Supplementary Table 1). The specimen will be housed at the Canadian Museum of Nature, Ottawa, Ontario, until such time as research and collections facilities are available in Nunavut.

**Diagnosis**. Elpistostegalian tetrapodomorph characterized by the following unique combination of characters: dorsoventral asymmetry in pectoral fin lepidotrichia (also present in *T. roseae*) and possession of a boomerang-shaped humerus lacking ventral ridge and associated foramina and ectepicondyle (distinct from *P. rhombolepis*, *E. watsoni*, *T. roseae* and more crownward tetrapods).

## Description

### Upper jaw and palate

**Fig. 2:**
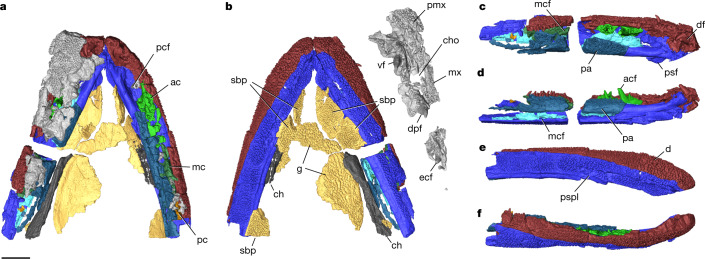
The feeding apparatus of *Q. wakei*. Volume renderings of µCT scans of the lower jaw and extra fragments reconstructed in their natural positions. **a**, Dorsal view of the lower jaws, ceratohyal, gular plates, premaxilla and palate. Scale bar, 1 cm. **b**, Ventral view with premaxillary and palatal elements displaced so ventral surfaces are visible. **c**, Left lower jaw, dorsal. **d**, Left lower jaw, medial. **e**, Right lower jaw, ventral. **f**, Right lower jaw, lateral. Abbreviations: ac, anterior coronoid; acf, anterior coronoid fang; ch, ceratohyal; cho, choana; d, dentary; df, dentary fang; dpf, dermopalatine fang; ecf, ectopterygoid fang; g, gulars; mc, Meckel’s cartilage; mcf, Meckelian canal foramen; mx, maxilla; pa, prearticular; pc, posterior coronoid; pcf, precoronoid fossa; pmx, premaxilla; pspl, postsplenial; sbp, submandibulo-branchiostegal plate; psf, post-symphyseal flange; vf, vomerine fang.

Rostral elements of the upper jaws and palate, including portions of the ectopterygoid, dermopalatine, vomer, premaxilla and maxilla are preserved (Fig. 2a,b, Extended Data Fig. 2 and Supplementary Video 2). These elements are primarily from the left side and preserved in articulation with the lower jaws. The vomer is broad, fanged and forms the posterior wall of the palatal fossa with a row of smaller teeth. Fangs and a row of smaller teeth are also present on the dermopalatine and ectopterygoid. An expanded mesial surface of the dermopalatine lacks teeth and overlaps slightly with the vomer, similar to *T. roseae*^8^, forming the mesial and posterior margin of the choana. The anterolateral wall of the choana is formed by a simple, smooth articulation of the premaxilla and maxilla. Maxillary teeth are smaller than the premaxillary teeth. In their respective tooth rows, maxillary and premaxillary teeth are uniform in size.

### Lower jaw

The lower jaws of *Q. wakei* are preserved in articulation anterior to the adductor chamber, including the dentary, infradentaries, coronoids and prearticular (Fig. 2). The symphysis is relatively smooth, not interdigitating. Large fangs with plicidentine infolding are present on the dentary, anterior coronoid and middle coronoid. Rows of smaller dentition are also present on the coronoids and dentary, including evidence of an auxiliary lateral tooth row on the dentary. The prearticular has a broad shagreen field of denticles that is raised adjacent to coronoids, and the denticles possess a distinct dorsoventral gradient in size. The adsymphyseal is missing, but small teeth embedded in the matrix of the precoronoid fossa suggest it was present in life.

Infradentaries are identifiable by the presence of the mandibular canal and postsplenial pit line. The mandibular canal is an open groove along most of its length, but in areas of the most intact preservation it takes the form of discrete pits the bone surface. The splenial has a larger postsymphyseal flange than in *T. roseae* but has a similar articulation with the prearticular^4^. Boundaries between the infradentaries are obscured by overlying dermal sculpting and are difficult to distinguish in computed tomography cross-section.

The Meckelian canal contains only partially ossified Meckelian bone along its length, but evidence of Meckelian ossification extends from the symphysis to the posterior coronoids. The canal is exposed lingually ventral to the prearticular and, in areas of intact ossification, Meckelian fenestra are bordered dorsally by Meckelian bone and ventrally by infradentaries.

### Gular plates and ceratohyal

Fragments of a principal and median gular plate are preserved, along with a series of submandibulo-branchiostegal plates (Fig. 2a,b). A straight, grooved ceratohyal lies immediately adjacent to the left lower jaw. A small anterior fragment of the right ceratohyal is preserved adjacent to the right lower jaw^19–21^.

### Pectoral fin

**Fig. 3:**
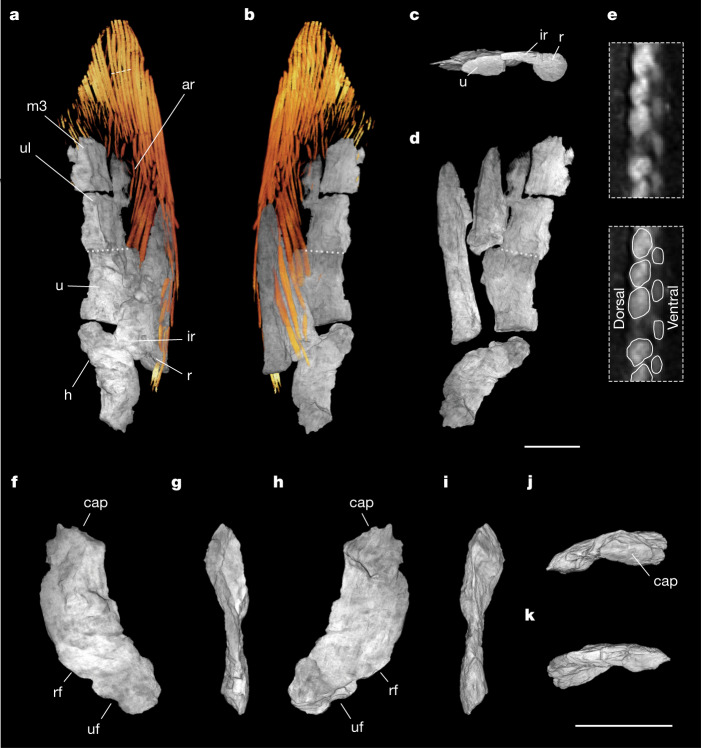
Left pectoral fin of *Q. wakei*. Volume renderings of µCT scans of the fin with scales removed. **a**,**b**, Dorsal (**a**) and ventral (**b**) views of the fin with endoskeleton in grey and dermal rays in orange. Dotted lines indicate the boundary between ulna and ulnare. The dashed line indicates position of cross-section in **e**, which is oriented orthogonal to the plane of the fin web. **c**, Endoskeleton viewed from the proximal side with humerus removed. **d**, Reconstruction of endoskeletal elements with estimated boundary between the radius and intermedium. Scale bar, 1 cm. **e**, Cross sections of the fin rays, showing asymmetry in the size of dorsal and ventral hemitrichia. **f**–**k**, Humerus in dorsal (**f**), pre-axial (anterior) (**g**), ventral (**h**), postaxial (posterior) (**i**), proximal (**j**) and distal (**k**) perspectives. Scale bar, 1 cm. Proximal is up in **f**–**i**. Dorsal is up in **j** and **k**. Abbreviations: ar, anterior radial; cap, caput humeri; h, humerus; ir, intermedium; r, radius; rf, radial facet; m3, third mesomere, u, ulna; ul, ulnare; uf, ulnar facet.

**Extended Data Fig. 3:**
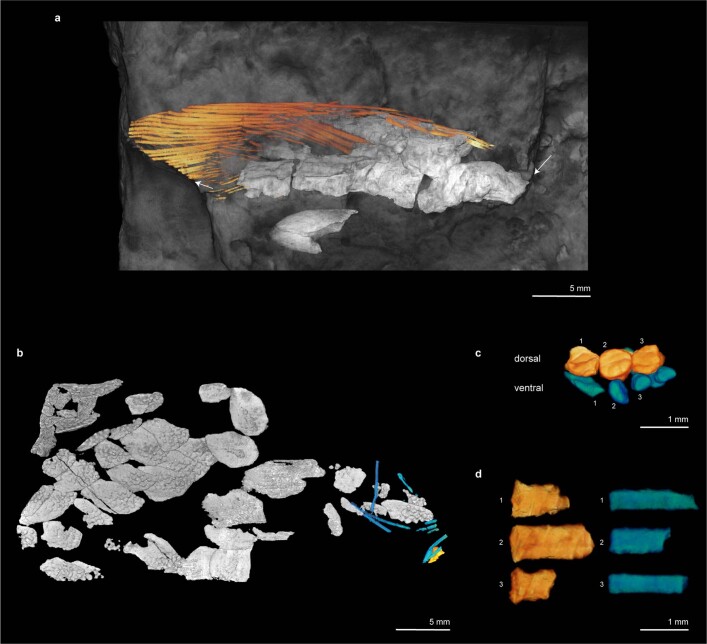
Additional fin-associated materials. (a) A thin, slightly convex bladelike element that might be part of the pectoral girdle is adjacent to the pectoral fin. Breaks in the block have exposed the proximal articular surface of the humerus and the posterodistal portion of the fin web. (b) An element, shown in Extended Data Fig. 2i, contains scales and additional lepidotrichia from a paired fin. (c) Fin rays, seen in the lower right corner of panel b, in their preserved position showing asymmetry between the dorsal and ventral hemitrichia. (d) Three pairs of hemitrichia from panel c repositioned and shown in dorsal perspective. Dorsal hemitrichia are orange, and ventral hemitrichia are blue.

The left pectoral fin includes the humerus, ulna, radius, intermedium, third mesomere, third radial, fin web and associated scales (Fig. 3a,b and Supplementary Video 3). The fin is embedded in matrix with the proximal articular surface of the humerus and the posterior distal fringe of the fin web exposed at the edges of the block (Extended Data Fig. 3a). Three endoskeletal elements contact the humerus. Two have robust proximal articular surfaces and are identified as the radius and ulna. The third, which lies between and slightly dorsal to them, is identified as the intermedium proximally displaced during preservation, although its shape is difficult to assess because of its position relative to other elements (Fig. 3c,d and Supplementary Discussion).

The fin is characterized by ventralward curvature of the radius and asymmetry in the lepidotrichia, in which dorsal hemitrichia have a greater cross sectional area than ventral hemitrichia, as in *T. roseae* (Fig. 3e and Extended Data Fig. 3c,d)^7^. Roughly 30 lepidotrichia are preserved. Similar to other finned tetrapodomorphs, rays are more robust anteriorly and more gracile posteriorly and rays are more terminally positioned on the posterior side^7^.

The humerus is boomerang-shaped and lacks numerous characteristic elpistostegalian features, notably a humeral ridge and associated foramina, ectepicondylar process, prominent entepicondyle and distinct articular surfaces for the ulna and radius (Fig. 3f–k). The ulna lacks a postaxial process and distally would have articulated with the intermedium and ulnare. The fin is gracile as compared to other elpistostegalians. The anteroposterior width of the humerus is narrower than the humeri of *T. roseae*^5^ and *E. watsoni*^9^ and more similar to *P. rhombolepis*^3^. The shallow dorsoventral depth of the fin might reflect compression; however, articular surfaces of the ulna and radius are similar in their geometry to three-dimensionally preserved specimens of *T. roseae* specimens (NUFV 108, 109, 110), suggesting that morphology was narrow in life (Supplementary Discussion).

### Scalation

**Extended Data Fig. 4:**
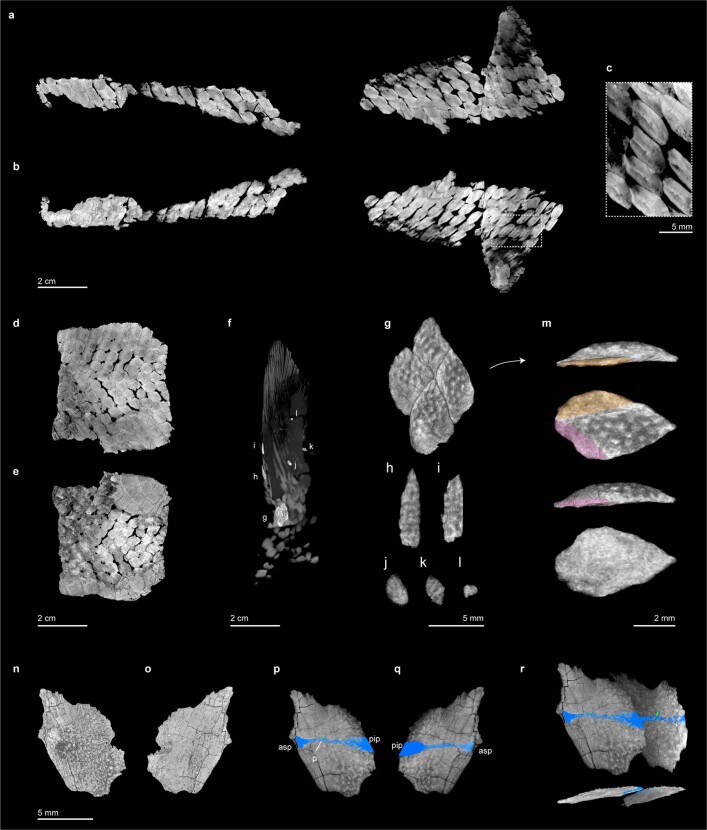
NUFV 137 scales. Internal (a) and external (b) views of scale field from left flank. (c) Scales outlined in panel b showing median ridge on internal surface. Internal (d) and external (e) views of scale field from dorsal midline. (f) Left pectoral fin in ventral aspect showing the position of individual scales figured in panels g-l. (g) Scales that covered the humerus ventrally. (h, i) Elongate scales from leading edge. (j–l) Small scales from the ventral surface of the fin. (m) One scale in pre-axial (anterior), external, post-axial (posterior) and internal views showing dermal sculpting and lack of ventral keel. (n, o) Left lateral line scale in external and internal views. (p, q) Scale with reduced opacity and the canal shown in blue. Midway along the length of the scale, a pore connects fluid in the canal and the external environment. (r) Two of the preserved lateral line scales in reconstructed position showing their degree of overlap and expected orientation relative to the epidermis. In panels m, n: area of overlap with adjacent scale in the row shown in orange, area of overlap with scale in adjacent row shown in pink. Abbreviations: asp, anterior suprascalar pore; pip, posterior infrascalar pore; p, pore.

Scales are preserved from the trunk, including dorsal midline and flank, the pectoral fin and the lateral line series (Extended Data Fig. 4). Scalation is broadly similar to other finned elpistostegalians^7,9,22^. Scales are rhomboid in shape with the free surface sculpted and a smooth internal surface that often bears a ventral keel (Extended Data Fig. 4a–c). On the trunk, scale rows extend posterolaterally from the dorsal midline, with individual scales partially covering the scale that follows in the row and also the scale of an adjacent posterior row (Extended Data Fig. 1d,e). Pectoral fin scales are smaller than those of the flank and show variation in their morphology (Extended Data Fig. 4f–m). Lateral line scales are preserved from the left flank and show a completely enclosed tube with anterior suprascalar and posterior infrascalar pores enlarged relative to the diameter of the canal, and a small pore midway along the length of the scale connecting the canal to the external environment (Extended Data Fig. 4n–r).

## Phylogenetic relationships

**Fig. 4:**
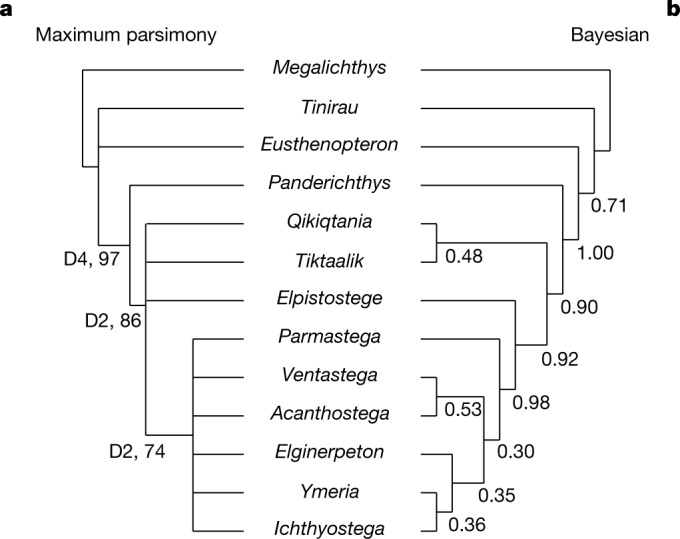
Phylogenetic analysis. **a**, Strict consensus tree from the maximum parsimony analysis with Bremer decay (D) and bootstrap support values. **b**, Majority-rule tree from undated Bayesian analysis with posterior probabilities. Both analyses recover a basal polytomy; *Megalichthys* is shown as the outgroup, consistent with other studies^9,23,25,36^.

The phylogenetic position of *Q. wakei* was analysed by maximum parsimony and undated Bayesian approaches, which were applied to a matrix of 13 taxa and 125 characters primarily assembled from previous publications (see Supplementary Information for detailed description of phylogenetic data)^9,23,24^. Both methods robustly recover *Q. wakei* as crownward to *P. rhombolepis* and, thus, as an elpistostegalian closely related to limbed tetrapods (Fig. 4). The analyses differ in their relative placement of *Q. wakei*, *T. roseae*, *E. watsoni*; a strict consensus tree of the 28 shortest trees recovered from maximum parsimony analyses shows an unresolved polytomy, whereas Bayesian analysis finds weak support for a sister relationship between *Q. wakei* and *T. roseae* with *E. watsoni* positioned more crownward. This is similar to other recent phylogenetic analyses of stem tetrapods, which have robustly recovered *Tiktaalik* and *Elpistostege* as outgroups to digited forms, although support for their relative positions is not strong^9,23,25^.

## Discussion

**Fig. 5:**
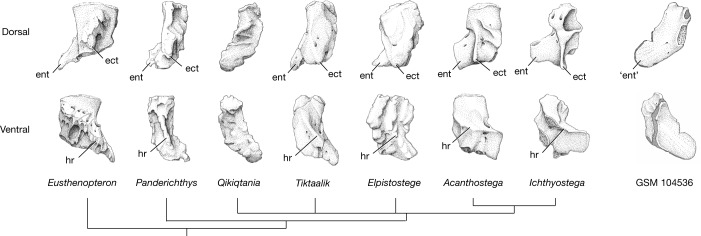
Humeri at the fin-to-limb transition. For consistency of orientation between species, several specimens have been reflected, so that each is represented as being from the right side. Illustrations are based on previously published descriptions: *Eusthenopteron*^28^, *Panderichthys*^2,3^, *Tiktaalik*^5^, *Elpistostege*^9^, *Acanthostega*^37^, *Ichthyostega*^30^ and GSM 104536 (refs. ^10,14^). Abbreviations: ect, ectepicondyle; ent, entepicondyle; hr, humeral or ventral, ridge.

*Q. wakei* reveals a combination of characters unique among stem tetrapods. The pectoral fin, lacking a postaxial process on the ulnare and showing accentuated hemitrichial asymmetry, is clearly elpistostegalian^5,7^. Yet, the morphology of the humerus is unlike others described. With the absence of a ventral ridge or ectepicondylar process and in possession of a general boomerang shape, it is more similar to the humerus previously attributed to the tetrapod, *Elginerpeton pancheni*^10^, than to any other Devonian taxon (Fig. 5). That specimen, GSM 104536, from Scat Craig in Scotland, is an isolated bone from a coeval deposit in Laurentia that generated debate as to whether it was from a tetrapod or whether it was even a humerus at all^10–14^. The similarity to *Q. wakei* suggests that GSM 104536 is indeed a humerus but belongs to a finned elpistostegalian, not a limbed tetrapod.

The morphology of the *Q. wakei* humerus is distinctive among stem tetrapods. Indeed, the lack of muscular processes on the humerus for flexors and extensors at the shoulder and elbow, the terminal position of the facets for the radius and ulna, and the relatively large surface area of the fin web suggest that the fin of *Q. wakei* is less suited for walking, trunk lifting and station holding in water than it is for a range of swimming behaviours^13^. With its gracile form and lacking many of the known main osteological correlates of muscular attachment^26^, the pectoral fin of *Q. wakei* represents a strategy of controlling hydrodynamic forces not seen in other stem tetrapods. As these features are not seen in tristichopterids, osteolepids or rhizodontids, they probably arose as apomorphies in elpistostegalians.

The holotype of *Q. wakei* is estimated to be a standard length of 75 cm (calculated from the proportions of *E. watsoni* specimen MHNM 06-2067 (ref. ^9^) scaled to the length of the lower jaw), making it smaller than other described elpistostegalians. The ontogenies of *Eusthenopteron foordi* and *T. roseae* provide evidence that, despite its relatively small size, the unique humeral morphology of *Q. wakei* reflects phylogenetic signal and not developmental stage. *E. foordi* individuals are described spanning more than 40-fold variation in size^27^, and across a broad range of sizes uniformly retain a ventral ridge, entepicondylar process and orientations of facets for articulation with the radius and ulna^28,29^. *T. roseae*, which is known from humeri ranging twofold in size, show a similar pattern, preserving these features across this size range, although overall proportions might vary^5,7^. Thus, major ontogenetic shifts in limb skeletal anatomy of *Ichthyostega* and *Acanthostega*, indicated to correspond to aquatic subadults transitioning to more terrestrial adult lifestyles using appendage-based substrate support, are derived for limbed forms^30^. Finned tetrapodomorphs, by contrast, are predicted to show more minor changes in the proportions of endoskeletal, and potentially dermal, components of their paired fins^7^.

With two elpistostegalian genera now known from nearby localities in Canadian Arctic and others from Quebec^9^, Latvia^31,32^ and potentially Russia^33^, Australia^34^ and Scotland^10^, the group probably has a wide distribution by the Frasnian Stage of the Late Devonian. This broad biogeographic range, coupled with the morphological disparity revealed by *Q. wakei*, hints at a wider diversity of elpistostegalians than known at present, with the closest relatives of tetrapods adapting in new ways to benthic, littoral and open water habitats by the Late Devonian^25,35^.

## Methods

### Computed tomography scanning

µCT scans were collected at The University of Chicago’s PaleoCT scanning facility using a GE Phoenix v|tome|x 240 kV/180 kV scanner (http://luo-lab.uchicago.edu/paleoCT.html). Scan parameters are reported in Supplementary Table 1. µCT data were reconstructed with Phoenix Datos|x 2 (v.2.3.3), imported to VGStudio Max (v.2.2) for cropping and exportation as a 16-bit tiff stack. Tiff stacks were segmented and visualized in Amira v.20.2 (FEI Software). For some scans, to accommodate for computational challenges that arise from large file sizes, data were converted to 8-bit files for segmentation; in such cases, after segmentation the renderings were generated from the original 16-bit files. Animations were generated by exportation tiff stacks from Amira and then edited with Adobe Premiere (v.13.12). High-resolution versions of images from all figures are provided in the Supplementary Data.

### Phylogenetic analyses

We investigated the phylogenetic position of *Q. wakei* using a phylogenetic data set of 13 taxa and 125 characters (see Supplementary Information for a detailed description of the phylogenetic matrix). All characters were treated as equally informative, and we assumed unordered evolution among states.

**Extended Data Fig. 5:**
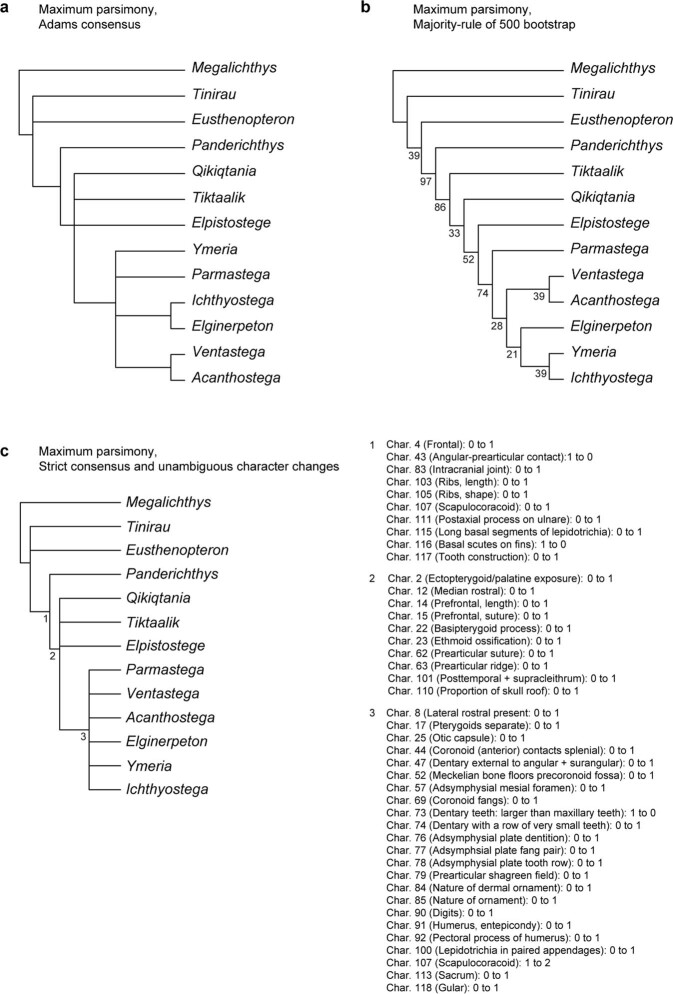
Expanded results of phylogenetic analyses. (a) Adams consensus tree of maximum parsimony analyses. (b) Majority rule tree of maximum parsimony analyses with bootstrapping (500 replicates). In all panels, *Megalichthys* is plotted as the outgroup consistent with previous phylogenetic analyses of early tetrapods^9,23,25,36^, although basal polytomies are recovered. (c) Unambiguous character changes recovered on the strict consensus tree using the command ‘apolist’ from PAUP*^38^.

Maximum parsimony analyses were performed using PAUP* (v.4.0a168)^38^. The branch and bound method for searching tree space was used with no topological constraints. A total of 28 most-parsimonious trees were recovered (tree length 151 s). The trees are summarized as a strict consensus tree (Fig. 4) and as an Adams consensus tree (Extended Data Fig. 5a). Clade support was estimated using two approaches: Bremer decay values^39^, calculated with AutoDecay (v.5.06)^40^ and non-parametric bootstrapping, calculated in PAUP* with 500 replicates (Fig. 4 and Extended Data Fig. 5b). Apomorphies of nodes in the strict consensus tree were identified using the function ‘apolist’ in PAUP*, which returns characters optimized under both accelerated transformation (ACCTRAN) and delayed transformation (DELTRAN) conditions (Extended Data Fig. 5c).

Undated Bayesian analyses were performed using MrBayes (v.3.2.7a)^41^. Analyses were run for five million generations with four runs of four chains sampling every 5,000 generations and a burn-in of 20%. *Megalichthys* was designated as an outgroup, consistent with other studies^9,23,25,36^.

Convergence was assessed with diagnostics reported by MrBayes (average s.d. of split frequencies <0.02, potential scale reduction factors was 1, effective sample sizes >200). Results are summarized by a majority-rule consensus tree of postburn-in trees (Fig. 4).

For both maximum parsimony and Bayesian analyses, executable files, log files, and individual trees that contribute to the summary trees are included as supplementary files (Supplementary Data). Custom R (v.3.6.1) code used for the calculation of Bremer decay values and for visualization of phylogenies are available at https://github.com/ThomasAStewart/Qikiqtania. All code is archived at Zenodo (10.5281/zenodo.6557684).

### Reporting summary

Further information on research design is available in the Nature Research Reporting Summary linked to this paper.

## Online content

Any methods, additional references, Nature Research reporting summaries, source data, extended data, supplementary information, acknowledgements, peer review information; details of author contributions and competing interests; and statements of data and code availability are available at 10.1038/s41586-022-04990-w.

## Supplementary information

Supplementary Information This file contains Phylogenetic Data; Supplementary Discussion; Supplementary Table 1; Captions for Videos S1 to S3; List of Supplementary Data Files and Supplementary References.

Reporting Summary.

Supplementary Video 1 Volumetric rendering of all NUFV 137 elements in approximate positions.

Supplementary Video 2 Volumetric rendering of the feeding apparatus of NUFV 137.

Supplementary Video 3 Volumetric rendering of the pectoral fin of NUFV 137.

Supplementary Data 1 A zipped file containing high-resolution images of all figures.

Supplementary Data 2 A zipped file that contains a PAUP* executable file, each of the most-parsimonious trees, and consensus trees (strict, Adams and 50% majority-rule).

Supplementary Data 3 A zipped file that contains a MrBayes executable file, screen log, and majority-rule consensus tree.

## Data Availability

All data are available for download. Computed tomography data sets and STL files of main elements are available for download from MorphoSource (https://www.morphosource.org/projects/000375542). Phylogenetic data are in Supplementary Data Files 2 and 3. Source data are provided with this paper.

## Extended data

are available for this paper at 10.1038/s41586-022-04990-w.

## Code availability

Executable files for PAUP* and MrBayes are available in the supplementary materials. Custom R code used for the calculation of Bremer decay values and for visualization of phylogenies are available at https://github.com/ThomasAStewart/Qikiqtania. All code is archived at Zenodo (10.5281/zenodo.6557684).
