# Supplementary material for: A new elpistostegalian from the Late Devonian of the Canadian Arctic
Source: Nature. 2022 Jul 20;608(7923):563–8. doi: 10.1038/s41586-022-04990-w (PMC9385497; doi:10.1038/s41586-022-04990-w)
Supplement: Supplementary file 1 — This file contains Phylogenetic Data; Supplementary Discussion; Supplementary Table 1; Captions for Videos S1 to S3; List of Supplementary Data Files and Supplementary References. [file 41586_2022_4990_MOESM1_ESM.pdf]

nature portfolio

https://doi.org/10.1038/s41586-022-04990-w

Supplementary information

A new elpistostegalian from the Late Devonian of the Canadian Arctic

In the format provided by the authors and unedited

nature portfolio

https://doi.org/10.1038/s41586-022-04990-w

Supplementary information

A new elpistostegalian from the Late Devonian of the Canadian Arctic

In the format provided by the authors and unedited

Supplementary Information for

A New Elpistostegalian from the Late Devonian of Canadian Arctic

T.A. Stewart[1,2], J.B. Lemberg[1], A. Daly[1], E.B. Daeschler[3], N.H. Shubin[1†]

[1]Department of Organismal Biology and Anatomy, University of Chicago; Chicago, USA.

[2]Current Address: Department of Biology, The Pennsylvania State University, State College, PA.

[3]Department of Vertebrate Zoology, Academy of Natural Sciences of Drexel University, Philadelphia, USA.

This PDF file includes:

Phylogenetic Data 2

Supplementary Discussion 17

Supplementary Table 1 19

Captions for Videos S1 to S3 21

List of Supplementary Data Files 22

Supplementary Information References 23

Phylogenetic Data

Taxonomic sampling for phylogenetic analyses

These data are primarily based upon phylogenetic analyses of early tetrapods by Ahlberg and Clack[23], which included data for 10 of the 12 previously described taxa in this study (*Acanthostega*, *Elginerpeton*, *Elpistostege*, *Eusthenopteron*, *Ichthyostega*, *Panderichthys*, *Parmastega*, *Tiktaalik*, *Ventastega*, and *Ymeria*). This taxon set was expanded to include data for two additional tetrapodomorphs, *Megalichthys* and *Tinirau*, using the phylogenetic matrixes of Swartz[24] and Cloutier et al.[9].

Character coding

Characters 1-109 are from Ahlberg and Clack[23]. Data for *Megalichthys* and *Tinirau* were added for these characters by manually matching the coding of characters from Swartz[24] and Cloutier et al.[9] as noted in the character list. Coding for *Megalichthys* was confirmed by checking species-level coding in Clement et al.[36].

Cloutier *et* al.[9] presented a phylogenetic analysis of tetrapodomorphs and in that work reevaluated and updated a number of previously published character codings. If any character that they updated was included amongst characters 1-109, we adopted their changes, with one exception, character 90 (the presence or absence of digits). We code *E. watsoni* as ambiguous for this character. Where Cloutier et al.[9] changes were applied to the characters of the Ahlberg and Clack[23] matrix, it has been noted below as ‘character changed’ with reference and description given.

Characters 110-121 are from Cloutier et al.[9]. The Cloutier paper included data from 9 of the 12 previously described taxa in this study (*Acanthostega*, *Elpistostege*, *Eusthenopteron*, *Ichthyostega*, *Tiktaalik*, *Panderichthys*, *Ventastega*, *Megalichthys* and *Tinirau*). For those not included in their data set (*Elginerpeton*, *Parmastega*, and *Ymeria*), we referred to the literature to evaluate whether coding could be added. For all instances where additional data is included for these three taxa, it is noted below as ‘coding added’ with references given.

Characters 122-125, which focus on post-cranial anatomy, are new characters. All instances of data being included for these four characters is noted below as ‘coding added’ with references given.

Character list

The source of each character is noted at the end of the character description:
AC -Ahlberg and Clack 2020 (largely from Clack and Ahlberg[42] and [43]);
C - Cloutier et al 2020[9];
S - Swartz 2012[24].

1 Anterior tectal/septomaxilla: anterior tectal (external bone, dorsal to nostril): = 0, septomaxilla (external or internal bone, posterior to nostril) = 1, absent = 2 (AC1, C5, S84)

2 Ectopterygoid/palatine exposure: more or less confined to tooth row = 0, broad mesial exposure additional to tooth row = 1 (AC2, S76)

3 Ectopterygoid reaches subtemporal fossa: no = 0, yes = 1 (AC3, S79)

4 Frontal: absent = 0, present = 1 (AC4, C19, S113)

5 Intertemporal: present = 0, absent = 1 (AC5, C16, S118)

6 Jugal: does not extend anterior to orbit = 0, extends anterior to orbit = 1 (AC6, C51, S94)

7 Lacrimal: contributes to orbital margin = 0, excluded from margin = 1 (AC7, C53, S92)

8 Lateral rostral present: yes = 0, no = 1 (AC8, S85)

9 Maxilla makes interdigitating suture with vomer: no = 0, yes = 1 (AC9, S55)

10 Maxilla external contact with premaxilla: narrow contact point not interdigitated = 0, interdigitating suture = 1 (AC10, S54)

11 Maxilla extends behind level of posterior margin of orbit: yes = 0, no = 1 (AC11)

12 Median rostral: single = 0, paired = 1, absent = 2 (AC12, S86)

13 Opercular: present = 0, absent = 1 (AC13, C113, S139)

14 Prefrontal: twice as long as broad, or less = 0, three times as long as broad or more = 1 (AC14, S106)

15 Prefrontal: transverse anterior suture with tectal = 0, tapers to point anteriorly = 1 (AC15, S107)

16 Preopercular: present = 0, absent = 1 (AC16, ~C58, S138)

17 Pterygoids separate in midline = 0, meet in midline anterior to cultriform process = 1 (AC17, C71, S70)

18 Pterygoid quadrate ramus margin in subtemporal fossa: concave = 0, with some convex component = 1 (AC18, 71)

19 Vomers separated by parasphenoid > half length: yes = 0, no = 1 (AC19, ~C67)

20 Vomers excluded from margin of interpterygoid vacuity: yes = 0, no = 1 (AC20)

21 Vomers nearly as broad as long, or broader = 0, about twice as long as broad, or longer = 1 (AC21, C61, S57)

22 Basipterygoid process: not strongly projecting with concave anterior face = 0, strongly projecting with flat anterior face = 1 (AC22, S12)

23 Ethmoid: fully ossified = 0, partly or wholly unossified = 1 (AC23, S1)

24 Hypophysial region: solid side wall pierced by small foramina for pituitary vein and other vessels = 0, single large foramen = 1 (AC24, S13)

25 Otic capsule: lateral commissure bearing hyomandibular facets: present = 0, absent = 1 (AC25, S14)

26 Parasphenoid: does not overlap basioccipital = 0, overlaps basioccipital =1 (AC26, S68)

27 Parasphenoid: denticulated field: present = 0, absent = 1 (AC27, S66)

28 Sphenoid: fully ossified, terminating posteriorly in intracranial joint or fused to otoccipital = 0, separated from otoccipital by unossified gap = 1 (AC28)

29 Ectopterygoid fang pairs: present = 0, absent = 1 (AC29, ~C73, S80)

30 Ectopterygoid row (3+) of smaller teeth: present = 0, absent = 1 (AC30, S81)

31 Ectopterygoid / palatine shagreen field: absent = 0, present = 1 (AC31, S78)

32 Maxilla tooth number: > 40 = 0, 30-40 = 1, < 30 = 2 (AC32)

33 Palatine row of smaller teeth: present = 0, absent = 1 (AC33)

34 Pterygoid shagreen: dense = 0, a few discontinuous patches or absent = 1 (AC34, S73)

35 Premaxillary tooth proportions: all approximately same size = 0, posteriormost teeth at least twice height of anteriormost teeth = 1 (AC35, ~C187, S53)

36 Vomerine fang pairs: present = 0, absent = 1 (AC36, S58)

37 Vomerine fang pairs noticeably smaller than other palatal fang pairs: no = 0, yes = 1 (AC37, S59)

38 Vomer anterior wall forming posterior margin of palatal fossa bears tooth row meeting in midline: yes = 0, no = 1 (AC38, S61)

39 Vomerine row of small teeth: present = 0, absent = 1 (AC39, S60)

40 Vomerine shagreen field: absent = 0, present = 1 (AC40, S62)

41 Adductor fossa faces dorsally = 0, mesially = 1 (AC41)

42 Adductor crest: absent = 0, peak anterior to adductor fossa, dorsal margin of fossa concave = 1, peak above anterior part of adductor fossa, dorsal margin of fossa convex = 2 (AC42, S52)

43 Angular-prearticular contact: prearticular contacts angular edge to edge = 0, absent = 1, mesial lamina of angular sutures with prearticular = 2 (AC43, ~C91, S48)

44 Coronoid (anterior) contacts splenial: no = 0, yes = 1 (AC44, C89, S40)

45 Coronoid (posterior) posterodorsal process: no = 0, yes = 1 (AC45, S40)

46 Coronoid (posterior) posterodorsal process visible in lateral view: no = 0, yes = 1 (AC46, S43)

47 Dentary external to angular + surangular, with chamfered ventral edge and no interdigitations: no = 0, yes = 1 (AC47)

48 Dentary ventral edge: smooth continuous line = 0, abruptly tapering or ‘stepped’ margin = 1 (AC48, S27)

49 Mandibular sensory canal: present = 0, absent = 1 (AC49, S131)

50 Mandibular canal exposure: entirely enclosed, opens through lines of pores = 0, mostly enclosed, short sections of open grooves = 1, mostly open grooves, short sections opening through pores = 2, entirely open = 3 (AC50, S132)

51 Mandible: oral sulcus/surangular pit line: present = 0, absent = 1 (AC51, S133)

52 Meckelian bone floors precoronoid fossa: yes = 0, no = 1 (AC52)

53 Meckelian bone ossified in middle part of jaw: yes = 0, little or no ossification = 1 (AC53, ~C78)

54 Meckelian foramina/ fenestrae, dorsal margins formed by; Meckelian bone = 0, prearticular = 1, infradentary = 2 (AC54, S31)

55 Meckelian foramina/ fenestrae, height: much lower than adjacent prearticular = 0, equal to or greater than depth of adjacent prearticular = 1 (AC55, S32)

56 Adsymphysial lateral foramen present: no = 0, yes = 1 (Following Ahlberg and Clack 2020: the character follows a terminology change from "parasymphysial" to "adsymphysial.") (AC56, S20)

57 Adsymphysial mesial foramen present: no = 0, yes = 1 (AC57, C96, S21)

58 Postsplenial with mesial lamina: no = 0, yes = 1 (AC58, S30)

59 Postsplenial pit line present: yes = 0, no = 1 (AC59)

60 Postsplenial suture with prearticular present: no = 0, yes but interrupted by Meckelian foramina or fenestrae = 1, uninterrupted suture = 2 (AC60, C88, S29)

61 Prearticular sutures with surangular: no = 0, yes = 1 (AC61, S49)

62 Prearticular sutures with mesial lamina of splenial: no, mesial lamina of splenial absent = 0, yes = 1, no, mesial lamina of splenial separated from prearticular by postsplenial = 2 (AC62, C90)

63 Prearticular with longitudinal ridge below coronoids: no = 0, yes = 1 (AC63, C102)

64 Prearticular with mesially projecting flange on dorsal edge along posterior border of adductor fossa: no = 0, yes = 1 (AC64, S51)

65 Prearticular centre of radiation of striations: level with posterior end of posterior coronoid = 0, level with middle of adductor fossa = 1, level with posterior end of adductor fossa = 2 (AC65)

66 Splenial has free ventral flange: yes = 0, no = 1 (AC66)

67 Splenial, rearmost extension of mesial lamina: closer to anterior end of jaw than to adductor fossa = 0, equidistant = 1, closer to anterior margin of adductor fossa than to the anterior end of the jaw = 2 (AC67, ~C90)

68 Coronoids: at least one has fang pair recognizable because at least twice the height of coronoid teeth: yes = 0, no = 1 (AC68, ~C97, S36)

69 Coronoids: at least one has fangs recognizable because noticeably mesial to vertical lamina of bone and to all other teeth: yes = 0, no = 1 (AC69)

70 Coronoids: at least one has organized tooth row: yes = 0, no =1 (AC70, ~C98, S38)

71 Coronoids: at least one carries shagreen: no = 0, yes = 1 (AC71, S37)

72 Coronoids: size of teeth (excluding fangs) on anterior and middle coronoids relative to dentary tooth size: about the same = 0, half height or less = 1 (AC72, S39)

73 Dentary teeth: larger than maxillary teeth = 0, same size as maxillary teeth = 1, smaller than maxillary teeth = 2 (AC73, S23)

74 Dentary with a row of very small teeth or denticles lateral to tooth row: yes = 0, no = 1 (AC74, C87, S24)

75 Adsymphysial tooth plate: present = 0, absent = 1 (AC75, C93, ~S16)

76 Adsymphysial plate dentition: shagreen or irregular tooth field = 0, organized dentition aligned parallel to jaw margin = 1, no dentition = 2 (AC76, ~C95, S17)

77 Adsymphsial plate has fang pair: no = 0, yes = 1 (AC77, S18)

78 Adsymphysial plate has tooth row: no = 0, short tooth row, separated from coronoid tooth row by diastema = 1, long tooth row reaching coronoid = 2 (AC78, ~C95)

79 Prearticular shagreen field, distribution: gradually decreasing from dorsal to ventral = 0, well defined dorsal longitudinal band = 1, scattered patches or absent = 2 (AC79, S50)

80 Anterior palatal fenestra: single = 0, double = 1, absent = 2 (AC80, S74)

81 Dorsal fontanelle on snout: absent = 0, present = 1 (AC81, S87)

82 Interpterygoid vacuities: absent = 0, at least 2 x longer than wide = 1, < 2 x longer than wide = 2 (AC82, S75)

83 Intracranial joint: present in dermal skull roof = 0, absent = 1 (AC83, C25, S119)

84 Nature of dermal ornament: tuberculate = 0, fairly regular pit and ridge = 1, irregular = 2, absent or almost absent = 3 (AC84, S195)

85 Nature of ornament: ‘starbursts’ of radiating ornament on at least some bones: no = 0, yes = 1 (AC85, S196)

86 Keyhole-shaped orbits: absent = 0, present = 1 (AC86)

87 Anocleithrum: oblong with distinct anterior overlap area = 0, drop-shaped with no anterior overlap area = 1, absent = 2 (AC87, C188, S147)

88 Cleithrum: ornamented = 0, not ornamented = 1 (AC88, C126, S197)

89 Cleithrum, postbranchial lamina: present = 0, absent = 1 (AC89, S149)

90 Digits: absent = 0, present = 1 (AC90, C152, S178)

91 Humerus: narrow tapering entepicondyle = 0, square or parallelogram-shaped entepicondyle = 1 (AC91, ~C145)

92 Pectoral process of humerus: absent = 0, present = 1 (AC82, C146)

93 Proximal limb of oblique ridge of humerus: present, separated from anterior margin of humerus by prepectoral space = 0, absent, replaced by deltopectoral crest = 1 (AC93)

94 Latissimus dorsi attachment of humerus: diffuse ridged area = 0, distinct process = 1 (AC94)

95 Foramina piercing oblique ventral ridge of humerus: many = 0, one moderately large foramen in addition to entepicondylar foramen = 1, entepicondylar foramen is the only large opening, other foramina are tiny pinpricks or absent = 2 (AC95)

96 Ilium, iliac canal: absent = 0, present = 1 (AC96, S180)

97 Ilium, posterior process: oriented posterodorsally = 0, oriented approximately horizontally posteriorly = 1 (AC97, S188)

98 Interclavicle: small and concealed or absent = 0, large and exposed = 1 (AC98, ~C134, S158)

99 Interclavicle shape: ovoid = 0, kite-shaped = 1, with posterior stalk = 2 (AC99, C190, S159)

100 Lepidotrichia in paired appendages: present = 0, absent = 1 (AC100, C194)

101 Posttemporal + supracleithrum: present = 0, absent = 1 (C101, C124, S144+S145)

102 Radius and ulna: radius much longer than ulna = 0, approximately equal length = 1 (AC102, C193)

103 Ribs, trunk: no longer than diameter of intercentrum = 0, longer = 1 (AC103, C195, S183)

104 Ribs, trunk: all straight = 0, at least some curving ventrally = 1 (AC104, S184)

105 Ribs, trunk: all cylindrical = 0, some or all bear flanges from posterior margin which narrow distally = 1, some or all flare distally = 2 (AC105, C196, S185)

106 Scapular blade: absent = 0, small with narrow top = 1, large with broad top = 2 (AC106, ~C136, S153)

107 Scapulocoracoid: small and tripodal = 0, large plate pierced by large coracoid foramen = 1, very large plate without large coracoid foramen = 2 (AC107, ~C135)

108 Subscapular fossa: broad and shallow = 0, deeply impressed posteriorly = 1 (AC108)

109 Squamation: complete body covering of scales, all similar = 0, ventral armour of gastralia = 1 (AC109, S200)

110 Proportion of skull roof lying anterior to middle of orbits: <50% = 0, >=50% = 1 (C2)

111 Postaxial process on ulnare: present = 0, absent = 1 (C147)

112 Radius length: longer than humerus = 0, equal to or shorter than humerus = 1 (C149)

113 Sacrum: absent = 0, present = 1 (C159)

114 Scales: round = 0, rhombic = 1 (C162)

115 Long basal segments of lepidotrichia in pectoral fin: absent = 0, present = 1 (C164)

116 Basal scutes on fins: absent = 0, present = 1 (C165)

117 Tooth construction: simple or generalized polyplocodont = 0, labyrinthodont = 1 (C169)

118 Gular: present = 0, absent = 1 (C177)

119 Olecranon process on ulna: absent = 0, present = 1 (C182)

120 Number radials articulating on ulnare 0-2 radials = 0, greater than 2 radials = 1 (C199)

121 Tabular horn: absent = 0, present = 1 (C202)

122 Dorsal fins: two = 0, fewer than two = 1 (new character)

123 Anal fin: present = 0, absent = 1 (new character)

124 Asymmetry in pectoral fin hemitrichia: cross sectional area (CSA) of hemitricha differ by less than 2-fold = 0, CSA is 2-fold or greater = 1 (new character)

125 Relative girdle size: pectoral girdle significantly taller than pelvic girdle in lateral aspect = 0, girdles are approximately the same height = 1 (new character)

Modified and new character codings

Acanthostega gunnari (3 codings added)

Character 122 was coded ‘1’ according to Coates[37] (their Fig. 7).

Character 123 was coded ‘1’ according to Coates[37] (their Fig. 7).

Character 125 was coded ‘1’ according to Coates[37] (their Figs. 14, 18, 19, 31).

Elginerpeton pancheni (1 coding added)

Character 113 was coded ‘1’ according to Ahlberg[10] (their char. 32).

Elpistostege watsoni (21 characters changed, 2 codings added)

Character 13 was changed from '?' to '0' on the basis of Cloutier et al.[9] (their char. 113)

Character 19 was changed from '?' to '1', on the basis of Cloutier et al.[9] (their char. 64)

Character 21 was changed from '?' to '0' on the basis of Cloutier et al.[9] (their char. 61)

Character 29 was changed from '?' to '0' on the basis of Cloutier et al.[9] (their char. 73)

Character 35 was changed from '0' to '1' on the basis Cloutier et al.[9] (their char.187)

Character 53 was changed from '?' to '0' on the basis of Cloutier et al.[9] (their char. 78)

Character 62 was changed from '?' to '0' on the basis of Cloutier et al.[9] (their char. 90)

Character 68 was changed from '?' to '0' on the basis of Cloutier et al.[9] (their char. 97)

Character 74 was changed from '?' to '1' on the basis of Cloutier et al.[9] (their char. 87)

Character 75 was changed from '?' to '0' on the basis of Cloutier et al.[9] (their char. 93)

Character 87 was changed from '?' to '0' on the basis of Cloutier et al.[9] (their char. 188)

Character 88 was changed from '?' to '1' on the basis of Cloutier et al.[9] (their char. 126)

Character 91 was changed from '?' to '0' on the basis of Cloutier et al.[9] (their char. 145).
Character 98 was changed from '?' to '1' on the basis of Cloutier et al.[9] (their char. 134).
Character 99 was changed from '?' to '1' on the basis of Cloutier et al.[9] (their char. 190).
Character 100 was changed from '?' to '0' on the basis of Cloutier et al.[9] (their char. 194).
Character 101 was changed from '?' to '1' on the basis of Cloutier et al.[9] (their char. 124).
Character 102 was changed from '?' to '0' on the basis of Cloutier et al.[9] (their char. 193).
Character 103 was changed from '?' to '0/1' on the basis of Cloutier et al.[9] (their char. 195).
Character 104 was coded as "?". Although Cloutier et al.[9] describe the pectoral fin of *E. watsoni* as possessing two digits, we regard this as uncertain. There are several reasons for this caution: (i) The position of the elements identified as the digits appears to be anterior to the primary axis of the fin, rather than positioned as a terminal series distal to the mesomeric axis. (ii) Multiple reconstructions are presented for the dataset that differ in the number, position, and geometry of the distal endoskeletal elements (their Fig. 3 c,d)[9]. (iii) The morphology of the elements is unusual for phalanges. Specifically, the anterior series has a distal phalanx with a proximal articular surface several times wider than the articular surface of its more proximal counterpart. The posterior series has a proximal phalanx with a post-axial flange that extends beyond the joint to nearly half the length of the more distal phalanx. To our knowledge, both patterns are unprecedented among digits. Given these matters of position, variable reconstruction, and unusual morphology, we regard the hypothesis that *E. watsoni* possessed digits as a valid one worthy of continued analysis; hence, the uncertainty in the coding.
Character 105 was changed from '?' to '1' on the basis of Cloutier et al.[9] (their char. 196).
Character 122 was coded '1' on the basis of Cloutier et al.[9] (their Fig. 1).
Character 123 was coded '0' on the basis of Cloutier et al.[9] (their Fig. 1).

Eusthenopteron foordi (4 codings added)
Character 122 was coded '0' on the basis of Andrews and Westoll[28] (their Fig. 23).
Character 123 was coded '0' on the basis of Andrews and Westoll[28] (their Fig. 23).
Character 124 was coded '0' on the basis of Stewart et al.[7] (their Fig. 5).
Character 125 was coded '0' on the basis of Andrews and Westoll[28] (their Fig. 23).

Ichthyostega (3 codings added)

Character 122 was coded ‘0’ on the basis of Ahlberg et al.[44] (their Fig. 1).
Character 123 was coded ‘0’ on the basis of Ahlberg et al.[44] (their Fig. 1).
Character 125 was coded ‘0’ on the basis of Ahlberg et al.[44] (their Fig. 1).

Megalichthys (9 codings changed, 2 codings added)
Character 53 was changed from ‘?’ to ‘0’ on the basis of Cloutier et al.[9] (their char. 78)
Character 62 was changed from ‘?’ to ‘0’ on the basis of Cloutier et al.[9] (their char. 90)
Character 63 was changed from ‘?’ to ‘0’ on the basis of Cloutier et al.[9] (their char. 102)
Character 91 was changed from ‘?’ to ‘0’ on the basis of Cloutier et al.[9] (their char. 145)
Character 92 was changed from ‘?’ to ‘0’ on the basis of Cloutier et al.[9] (their char. 146)
Character 100 was changed from ‘?’ to ‘0’ on the basis of Cloutier et al.[9] (their char. 194)
Character 101 was changed from ‘?’ to ‘0’ on the basis of Cloutier et al.[9] (their char. 124)
Character 102 was changed from ‘?’ to ‘0’ on the basis of Cloutier et al.[9] (their char. 193)
Character 107 was changed from ‘?’ to ‘0’ on the basis of Cloutier et al.[9] (their char. 135)
Character 122 was coded ‘1’ according to Wellburn[45] (their Plate XIII).
Character 123 was coded ‘1’ according to Wellburn[45] (their Plate XIII).

Panderichthys pancheni (3 characters changed)
Character 6 was changed from '0' to '0/1' on the basis of Cloutier et al.[9] (their char. 51)
Character 35 was changed from '0' to '1' on the basis of Cloutier et al.[9] (their char. 187)
Character 99 was changed from '?' to '0' on the basis of Cloutier et al.[9] (their char. 190)

Parmastega aelidae (2 codings added)
Character 118 was coded as ‘1’ on the basis of Beznosov et al.[46] (their discussion section).
Character 121 was added as ‘1’ on the basis of Beznosov et al.[46] (their Fig. 1 G).

Tiktaalik roseae (45 characters changed, 3 codings added)
We corrected and updated character codings for ~35% of the *T. roseae* data. These are based upon studies of the cranium[8,47], pectoral girdle and fins[7,48], pelvic girdle and fin[6]. When the anatomy has been figured, we refer to the pertinent manuscript and figure. If the character has not been figured but can be observed in a publicly available data set, we refer to that, providing a DOI of the dataset.

Character 1 was changed from '?' to '0' on the basis of Lemberg et al.[8], which describes the presence of an anterior tectal. They are diagnosable in CT scans of specimens NUFV 108, NUFV 110, and NUFV 149 and lie immediately anterior to the prefrontal and are overlapped slightly by the anterior tip of the lacrimal (data available here https://doi.org/10.17602/M2/M168208).

Character 2 was changed from '0' to '1' on the basis of Lemberg et al.[8], which shows the feature on specimen NUFV 108 (their Fig. 2 A).

Character 3 was changed from '?' to '1' on the basis of Lemberg et al.[8], which shows the feature on specimen NUFV 108 (their Fig. 2 A, B).

Character 5 was changed from '1' to '0' on the basis of Lemberg et al.[8], which shows the feature on specimen NUFV 108 (their Fig. 2 B).

Character 7 was changed from '?' to '1' on the basis of Lemberg et al.[8], which shows the feature on specimens NUFV 108 and NUFV 110 (their Figs. 1, 2B).

Character 8 was changed from '?' to '0' on the basis of Lemberg et al.[8], which presents CT data for specimens NUFV 108, NUFV 110 and NUFV 149 that show the presence of the lateral rostral (data available here https://doi.org/10.17602/M2/M168208).

Character 9 was changed from '?' to '0' on the basis of Lemberg et al.[8], which shows the feature on specimen NUFV 108 (their Fig. 2 A).

Character 20 was changed from '?' to '-' on the basis of CT data presented in Lemberg et al.[8] (data available here https://doi.org/10.17602/M2/M168208).

Character 21 was changed from '?' to '0' on the basis of Lemberg et al.[8], which shows the feature on specimen NUFV 108 (their Fig. 2 A).

Character 22 was changed from '0' to '1' on the basis of Lemberg et al.[8], which shows the feature on specimen NUFV 108 (their Fig. 2 A, B; Fig. 3A).

Character 23 was changed from '0' to '1' on the basis of Lemberg et al.[8], which presents CT data that show the ethmoid to be partially ossified. This is diagnosable in the scans, as the ethmoid shows a cortex of higher density ossification with more medial portions less fully ossified. These medial portions are also less ossified than either the lower jaw or vomer. This is observed most clearly in specimen NUFV 149 (data available here https://doi.org/10.17602/M2/M168955, https://doi.org/10.17602/M2/M168954)

Character 24 was changed from '?' to '0' on the basis of Downs et al.[47] (Fig 2). CT data presented in Lemberg et al.[8] for specimens NUFV 108, NUFV 110, and NUFV 149 support this diagnosis.

Character 27 was changed from '?' to '0' on the basis of Lemberg et al.[8], which shows the feature on specimen NUFV 108 (their Fig. 2 A).

Character 29 was changed from '?' to '0' on the basis of Lemberg et al.[8], which shows the feature on specimen NUFV 108 (their Fig. 2 A).

Character 30 was changed from '?' to '0' on the basis of Lemberg et al.[8], which shows the feature on specimen NUFV 108 (their Fig. 2 A) .

Character 31 was changed from '?' to '0' on the basis of Lemberg et al.[8], which shows the feature on specimen NUFV 108 (their Fig. 2 A).

Character 33 was changed from '?' to '0' on the basis of Lemberg et al.[8], which shows the feature on specimen NUFV 108 (their Fig. 2 A).

Character 36 was changed from '?' to '0' on the basis of Lemberg et al.[8], which shows the feature on specimen NUFV 108 (their Fig. 2 A).

Character 37 was changed from '?' to '0' on the basis of Lemberg et al.[8], which shows the feature on specimen NUFV 108 (their Fig. 2 A).

Character 38 was changed from '?' to '0' on the basis of Lemberg et al.[8], which shows the feature on specimen NUFV 108 (their Fig. 2 A).

Character 39 was changed from '?' to '0' on the basis of Lemberg et al.[8], which presents CT data for specimen NUFV 108 (their Fig. 2 A).

Character 40 was changed from '?' to '0' on the basis of Lemberg et al.[8], which presents CT data for specimen NUFV 108 (their Fig. 2 A).

Character 43 was changed from '0' to '0/1' on the basis of CT data of specimen NUFV 108, which was published in association with Lemberg et al.[8] (data available here: https://doi.org/10.17602/M2/M168208)

Character 51 was changed from '?' to '0' on the basis of CT data of specimen NUFV 108, which was published in association with Lemberg et al.[8] (data available here: https://doi.org/10.17602/M2/M168208)

Character 53 was changed from '0' to '0/1' on the basis of CT data of specimen NUFV 108, which was published in association with Lemberg et al.[8] (data available here: https://doi.org/10.17602/M2/M168208)

Character 54 was changed from '?' to '0' on the basis of CT data of specimen NUFV 108, which was published in association with Lemberg et al.[8] (data available here: https://doi.org/10.17602/M2/M168208)

Character 59 was changed from '?' to '0' on the basis of CT data of specimen NUFV 108, which was published in association with Lemberg et al.[8] (data available here: https://doi.org/10.17602/M2/M168208)

Character 60 was changed from '?' to '0' on the basis of CT data of specimen NUFV 108, which was published in association with Lemberg et al.[8] (data available here: https://doi.org/10.17602/M2/M168208)

Character 62 was changed from '0' to '1' on the basis of CT data of specimen NUFV 108, which was published in association with Lemberg et al.[8] (data available here: https://doi.org/10.17602/M2/M168208)

Character 63 was changed from '0' to '1' on the basis of CT data of specimen NUFV 108, which was published in association with Lemberg et al.[8] (data available here: https://doi.org/10.17602/M2/M168208)

Character 65 was changed from '?' to '-' on the basis of CT data of specimen NUFV 108, which was published in association with Lemberg et al.[8] (data available here: https://doi.org/10.17602/M2/M168208)

Character 66 was changed from '?' to '0' on the basis of CT data of specimen NUFV 108, which was published in association with Lemberg et al.[8] (data available here: https://doi.org/10.17602/M2/M168208)

Character 67 was changed from '?' to '0' on the basis of CT data of specimen NUFV 108, which was published in association with Lemberg et al.[8] (data available here: https://doi.org/10.17602/M2/M168208)

Character 73 was changed from '?' to '1' on the basis of CT data of specimen NUFV 108, which was published in association with Lemberg et al.[8] (data available here: https://doi.org/10.17602/M2/M168208)

Character 74 was changed from '1' to '0' on the basis of CT data of specimen NUFV 108, which was published in association with Lemberg et al.[8] (data available here: https://doi.org/10.17602/M2/M168208)

Character 76 was changed from '?' to '0' on the basis of Lemberg et al.[8], which presents CT data for specimen NUFV 108 (their Fig. 2 A).

Character 77 was changed from '?' to '0' on the basis of CT data of specimen NUFV 108, which was published in association with Lemberg et al.[8] (data available here: https://doi.org/10.17602/M2/M168208)

Character 79 was changed from '0' to '0/1' on the basis of CT data of specimen NUFV 108, which was published in association with Lemberg et al.[8] (data available here: https://doi.org/10.17602/M2/M168208)

Character 80 was changed from '?' to '0' on the basis of CT data of specimen NUFV 108, which was published in association with Lemberg et al.[8] (data available here: https://doi.org/10.17602/M2/M168208)

Character 95 was changed from '?' to '0' on the basis of Shubin et al.[5], which describes the humerus of *Tiktaalik* and shows the feature on specimen NUFV 109 (their Fig. 2). Stewart *et* al.[7], also presents CT data of the humerus of specimen NUFV 110 (their Fig 3, Video S3).

Character 99 was changed from '?' to '0' on the basis of Shubin et al.[48], which describe the interclavicles of specimen NUFV 109 (their Fig. 4.6).

Character 104 was changed from '0' to '1' on the basis of the specimen NUFV 108, which shows ventralward curvature of the posterior-most rib preserved on the left side.

Character 105 was changed from '?' to '1' on the basis of Daeschler et al.[4], which describes ribs in specimen NUFV 108 (their Figs. 3C, 6). Additional photographs of the ribs of NUFV 108 are provided in Shubin et al.[6] (their Fig. 2).

Character 108 was changed from '?' to '0' on the basis of Shubin et al.[5], which describes the shoulder girdle of specimen NUFV 112 (their Figs. 3, 5b).

Character 109 was changed from '?' to '0' on the basis of Daeschler et al.[4] (their Fig. 2) and Shubin et al.[6] (their Fig. 2), which show scalation on the dorsal and ventral surfaces, respectively, of specimen NUFV 108.

Character 122 was coded as ‘1’ on the basis of examination of the specimen NUFV 108. The specimen preserves the dorsal series of scales in position from posterior to the cranium to the pelvis. In other tetrapodomorphs where two dorsal fins are present (e.g., *Eusthenopteron*) the anterior dorsal fin is positioned anterior to or at the level of the pelvis. Therefore, we diagnose a condition of not having two dorsal fins. Whether a single dorsal fin posterior to the pelvis was present is unclear.

Character 123 was coded as ‘1’ on the basis of examination of the specimen NUFV 108, which preserves the axial skeleton and ventral scales posterior to the pelvis and does not preserve an anal fin.

Character 124 was coded as ‘1’ on the basis of Stewart et al.[7], which describes the anatomy of pectoral fin hemitrichia in specimens NUFV 108 and NUFV 109 (their Figs. 3, 5, S6).

Character 125 was coded as ‘1’ on the basis of Shubin et al.[6], which describes the right pelvis of specimen NUFV 108 (their Figs. 3, 5).

Tinirau clackae (6 character changed, 2 codings added)

Character 6 was changed from '0' to '?' on the basis of Cloutier et al.[9] (their char. 51).

Character 53 was changed from ‘?’ to ‘1’ on the basis of Cloutier et al.[9] (their char. 78)

Character 62 was changed from ‘?’ to ‘0’ on the basis of Cloutier et al.[9] (their char. 90)

Character 91 was changed from ‘?’ to ‘0’ on the basis of Cloutier et al.[9] (their char. 145)

Character 100 was changed from ‘?’ to ‘0’ on the basis of Cloutier et al.[9] (their char. 194)

Character 102 was changed from ‘?’ to ‘0’ on the basis of Cloutier et al.[9] (their char. 193)

Character 123 was coded ‘1’ according to Swartz[24] (their Fig. 2).

Character 125 was coded ‘1’ according to Swartz[24] (their Fig 2).

Ventastega curonica (2 characters changed)

Character 13 was changed from '1' to '?' on the basis of Cloutier et al.[9] (their char. 113).

Character 21 was changed from '0' to '?' on the basis of Cloutier et al.[9] (their char. 61).

Supplementary Discussion

Size and body proportions

Figure 1 c shows NUFV 137 framed by a line drawing of a body. This drawing is based upon the proportions of *E. watsoni* (specimen MHNM 06-2067[9]) and scaled to the length of the lower jaw. Assuming these proportions, NUFV 137 measures approximately 75 cm standard length (from tip of the snout to the end of the last vertebrae).

Taphonomy

The pectoral fin shows postmortem displacement of several elements. In other finned tetrapodomorphs, lepidotrichia of the pectoral fin do not extend further proximally than to the base of the radius. However, in NUFV 137 lepidotrichia are positioned more proximally, overlapping the humerus on the ventral side, indicating that the fin web has been shifted relative to the proximal endoskeleton.

The intermedium is also displaced—as preserved, it contacts the humerus proximally and is positioned slightly dorsal to the radius. Although it is difficult to discern the natural boundaries of the intermedium and the radius from cross sections of CT data alone, we estimated the boundaries of this element on the basis of external geometry of the fully segmented endoskeleton. The posterior boundary of the intermedium is clearly demarcated by the ulna, which is significantly deeper than the adjacent intermedium. The anterior boundary of the intermedium is more challenging to determine, as there is not an abrupt change in depth to denote the posterior margin of the radius. Because the distal extent of the intermedium is estimated to reach the distal terminus of the ulna in its preserved position, we approximated the anterior boundary of the intermedium so that the there was a gradual curve from the proximo-anterior corner to the postero-distal corner. On the basis of the geometry of the proximal articular surfaces of the radius and ulna, we demarcate the proximal width of the intermedium (Fig. 3 c). This width is consistent with the space available for articulation on the ulna (Fig. 3 d). We note that these reconstructions do not affect the diagnosis, phylogenetic analysis, or interpretations of *Q. wakei*.

The humerus is narrow in the dorsoventral direction, raising the question of the extent to which its morphology reflects dorsoventral compression. The posterodistal portion of the humerus that

articulates with the ulna is of a similar depth as the proximal articular surface of the ulna (Video S3), indicating that among the endoskeletal elements, the humerus is not disproportionately flattened. Given that the proximal articular surfaces of the radius and ulna (Fig. 3 c) are similar in their shape to other exceptionally three-dimensionally preserved tetrapodomorph humeri (e.g., *Sauripterus talori*[7,49] and *T. roseae*[7]), we argue that the much of the narrowness of the humerus reflects a gracile phenotype in life. We additionally note that such compression is unlikely to impact diagnosis of phylogenetic characters that are based on the fin. For example, both *P. rhombolepis* and *T. roseae* are known from multiple specimens showing degrees of dorsoventral compression (e.g., specimens GIT434-1[2] and PIN 3547-19[3] for *P. rhombolepis*, and specimens NUFV 109[5] and NUFV 110[7] for *T. roseae*). For both taxa, even in the compressed specimens features like ectepicondyle, humeral ridge and its associated foramina are preserved[2,3,5,7]. Similarly, the *E. watsoni* specimen MHNM 06-2067[9] is described as compressed, and its humerus preserves features that are absent in *Q. wakei*.

Table S1. µCT scanning parameters.

Each row represents an individually scanned element with voltage, current, filter, and resolution provided. All scans were collected using a GE Phoenix v|tome|x 240 kv/180kv scanner. All data are deposited on MorphoSource (https://www.morphosource.org/projects/000375542). Panel labels for each element correspond to photos in Extended Data Fig. 2.

panel	element	tube	voltage	current	filter	voxel size	DOI
a	symphysis	180	160 kV	60 µA	0.12 mm Cu	31.754 µm	https://doi.org/10.17602/M2/M407134
b	middle section of left jaws (lower and upper)	180	90 kV	108 µA	none	9.708 µm	https://doi.org/10.17602/M2/M408179
c	fragmentary portions of dermopalatine, ectopterygoid, middle coronoid and dentary	180	90 kV	200 µA	none	9.098 µm	https://doi.org/10.17602/M2/M408195
d	left principal gular and ceratohyal	180	90 kV	200 µA	none	18.337 µm	https://doi.org/10.17602/M2/M408201
e	fragmentary portions of palate and lower jaw	180	90 kV	105 µA	none	9.515 µm	https://doi.org/10.17602/M2/M408209
f	small posterior jaw fragment	180	90 kV	200 µA	none	9.265 µm	https://doi.org/10.17602/M2/M408289
g	fragment of the marginal tooth row	240	150 kV	350 µA	0.56 mm Sn	62.081 µm	https://doi.org/10.17602/M2/M408295
h	left pectoral fin	240	90 kV	380 µA	0.25 mm Cu	43.287 µm	https://doi.org/10.17602/M2/M408301

i	fragment containing fin rays and scales	180	90 kV	200 µA	none	21.555 µm	https://doi.org/10.17602/M2/M408306
j	small, crushed endochondral element	180	90 kV	200 µA	none	14.037 µm	https://doi.org/10.17602/M2/M410039
k	small vascularized endochondral element	180	90 kV	200 µA	none	8.342 µm	https://doi.org/10.17602/M2/M410051
l	small section of dorsal midline scales	240	100 kV	350 µA	none	35.096 µm	https://doi.org/10.17602/M2/M408312
m	small section of left lateral line scales	180	90 kV	115 µA	none	10.831 µm	https://doi.org/10.17602/M2/M408318
n	large section of left flank scales	240	100 kV	400 µA	none	59.004 µm	https://doi.org/10.17602/M2/M408324

Captions for Supplementary Video Files

Supplementary Video 1.

Volumetric rendering of all NUFV 137 elements in approximate positions.

Supplementary Video 1.

Volumetric rendering of the feeding apparatus of NUFV 137.

Supplementary Video 2.

Volumetric rendering of the pectoral fin of NUFV 137.

List of Supplementary Data Files

Supplementary Data 1. Image Files.

A zipped file containing high-resolution images of all figures.

Supplementary Data 2. PAUP* files.

A zipped file that contains a PAUP* executable file, each of the most-parsimonious trees, and consensus trees (strict, Adams and 50% majority-rule).

Supplementary Data 3. MrBayes files.

A zipped file that contains a MrBayes executable file, screen log, and majority-rule consensus tree.

Supplementary Information References

42 Clack, J. A. & Ahlberg, P. E. in *Recent Advances in the Origin and Radiation of Vertebrates* (eds G. Arratia, M.V.H. Wilson, & R. Cloutier) (Verlag Dr. Friedrich Pfeil, 2004).

43 Chen, D. *et al.* A partial lower jaw of a tetrapod from “Romer's Gap”. *Earth and Environmental Science Transactions of the Royal Society of Edinburgh* **108**, 55-65, doi:10.1017/S1755691018000099 (2017).

44 Ahlberg, P. E., Clack, J. A. & Blom, H. The axial skeleton of the Devonian tetrapod *Ichthyostega*. *Nature* **437**, 137-140, doi:http://www.nature.com/nature/journal/v437/n7055/suppinfo/nature03893_S1.html (2005).

45 Wellburn, E. D. On the Genus *Megalichthys*, Agassiz: Its History, Systematic Position, and Structure. *Proceedings of the Yorkshire Geological Society* **14**, 52–71 (1900).

46 Beznosov, P. A., Clack, J. A., Lukševičs, E., Ruta, M. & Ahlberg, P. E. Morphology of the earliest reconstructable tetrapod *Parmastega aelidae*. *Nature* **574**, 527-531, doi:10.1038/s41586-019-1636-y (2019).

47 Downs, J. P., Daeschler, E. B., Jenkins, F. A. & Shubin, N. H. The cranial endoskeleton of *Tiktaalik roseae*. *Nature* **455**, 925-929, doi:10.1038/nature07189 (2008).

48 Shubin, N. H., Daeschler, E. B. & Jenkins, F. A. in *Great Transformations in Vertebrate Evolution* (eds K.P. Dial, N.H. Shubin, & E.L. Brainerd) 63-76 (The University of Chicago Press, 2015).

49 Davis, M. C., Shubin, N. & Daeschler, E. B. A new specimen of *Sauripterus taylori* (Sarcopterygii, Osteichthyes) from the Famennian Catskill Formation of North America. *Journal of Vertebrate Paleontology* **24**, 26-40, doi:10.1671/1920-3 (2004).
