# Supplementary material for: A new elpistostegalian from the Late Devonian of the Canadian Arctic
Source: Nature. 2022 Jul 20;608(7923):563–8. doi: 10.1038/s41586-022-04990-w (PMC9385497; doi:10.1038/s41586-022-04990-w)
Supplement: Supplementary file 2 — Reporting Summary. [file 41586_2022_4990_MOESM2_ESM.pdf]

nature portfolio

Corresponding author(s): Neil Shubin

Last updated by author(s): May 19, 2022

Reporting Summary

Nature Portfolio wishes to improve the reproducibility of the work that we publish. This form provides structure for consistency and transparency in reporting. For further information on Nature Portfolio policies, see our Editorial Policies and the Editorial Policy Checklist.

Statistics

For all statistical analyses, confirm that the following items are present in the figure legend, table legend, main text, or Methods section.

n/a	Confirmed	
☒	☐	The exact sample size (*n*) for each experimental group/condition, given as a discrete number and unit of measurement
☒	☐	A statement on whether measurements were taken from distinct samples or whether the same sample was measured repeatedly
☒	☐	The statistical test(s) used AND whether they are one- or two-sided<br>*Only common tests should be described solely by name; describe more complex techniques in the Methods section.*
☒	☐	A description of all covariates tested
☒	☐	A description of any assumptions or corrections, such as tests of normality and adjustment for multiple comparisons
☒	☐	A full description of the statistical parameters including central tendency (e.g. means) or other basic estimates (e.g. regression coefficient) AND variation (e.g. standard deviation) or associated estimates of uncertainty (e.g. confidence intervals)
☒	☐	For null hypothesis testing, the test statistic (e.g. *F*, *t*, *r*) with confidence intervals, effect sizes, degrees of freedom and *P* value noted<br>*Give P values as exact values whenever suitable.*
☒	☐	For Bayesian analysis, information on the choice of priors and Markov chain Monte Carlo settings
☒	☐	For hierarchical and complex designs, identification of the appropriate level for tests and full reporting of outcomes
☒	☐	Estimates of effect sizes (e.g. Cohen's *d*, Pearson's *r*), indicating how they were calculated

Our web collection on statistics for biologists contains articles on many of the points above.

Software and code

Policy information about availability of computer code

Data collection	CT data were collected on a GE Phoenix v|tome|x 240 kv/180 kv scanner, reconstructed with Phoenix Datos|x 2 (v2.3.3), and then imported to VGStudio Max (v2.2) for cropping and exportation as a 16-bit tiff stack.
Data analysis	Tiff stacks were segmented and visualized in Amira v20.2 and Adobe Premiere (v13.12). Code for PAUP*(v4.0a168) and MrBayes (v3.2.7a) analyses are available as Supplementary Data Files 2 and 3, respectively. Custom R (v3.6.1) code used to plot phylogenetic trees are available on github (https://github.com/ThomasAStewart/Qikiqtania). All code is archived at Zenodo (DOI: 10.5281/zenodo.6557684).

For manuscripts utilizing custom algorithms or software that are central to the research but not yet described in published literature, software must be made available to editors and reviewers. We strongly encourage code deposition in a community repository (e.g. GitHub). See the Nature Portfolio guidelines for submitting code & software for further information.

Data

Policy information about availability of data

All manuscripts must include a data availability statement. This statement should provide the following information, where applicable:

- Accession codes, unique identifiers, or web links for publicly available datasets
- A description of any restrictions on data availability
- For clinical datasets or third party data, please ensure that the statement adheres to our policy

All data are available for download. Computed tomography data sets and STL files of major elements are available for download from MorphoSource (https://www.morphosource.org/projects/000375542). Phylogenetic data are provided in Supplementary Data Files 2 and 3.

Field-specific reporting

Please select the one below that is the best fit for your research. If you are not sure, read the appropriate sections before making your selection.

☐ Life sciences ☐ Behavioural & social sciences ☒ Ecological, evolutionary & environmental sciences

For a reference copy of the document with all sections, see nature.com/documents/nr-reporting-summary-flat.pdf

Ecological, evolutionary & environmental sciences study design

All studies must disclose on these points even when the disclosure is negative.

Study description	The study considers a new fossil specimen, NUFV 137. The specimen was CT scanned to reveal its anatomy. Phylogenetic data, based upon published studies and the new CT data, were analyzed to understand the evolutionary relationships of early tetrapods. The fossil will be housed to the Canadian Museum of Nature until such time as research and collections facilities are available within Nunavut.
Research sample	This study analyzes specimen NUFV 137. It is the only specimen collected from site NV0401. We describe its collection and its morphology.
Sampling strategy	There is only one known specimen (NUFV 137) of Qikiqtania wakei, which we describe in this paper. All fragments have been CT scanned, analyzed, and are presented in the study.
Data collection	Computed tomography data were collected at the University of Chicago's PaleoCT scanning facility. The scanner used was a GE Phoenix v|tome|x 240 kv/180 kv scanner. Data were collected by authors TAS and JBL.
Timing and spatial scale	CT data were collected between Jan. 2020 and Sept. 2022 according to machine availability.
Data exclusions	No data were excluded from analyses.
Reproducibility	There is only one known specimen (NUFV 137) of Qikiqtania wakei; therefore, concerns of replication are not applicable to this study.
Randomization	There is only one known specimen (NUFV 137) of Qikiqtania wakei; therefore, concerns of randomization are not applicable to this study.
Blinding	There is only one known specimen (NUFV 137) of Qikiqtania wakei; therefore, concerns of blinding are not applicable to this study.

Did the study involve field work? ☒ Yes ☐ No

Field work, collection and transport

Field conditions	High Arctic desert with average temps 0 centigrade with continual wind and occasional snow and sleet.
Location	Bird Fiord, Ellesmere Island Canada. Coordinates of site: N77°10.235' W86°11.279'.
Access & import/export	Fieldwork done with permits from Nunavut Research Board; Ministry of Culture, Heritage and Youth, Nunavut; and support from the Polar Continental Shelf Program of Canada. Export permit provided by Canadian Museum of Nature, working on behalf of the Nunavut Territory. Field permission was granted by the Hamlet of Grise Fiord, Canada; the Iviq Hunter and Trappers of Grise Fiord Canada.
Disturbance	Disturbances related to campsites include circles of rocks for tents as well as inorganic and organic waste. Surface disturbance at the fossil site includes a 8 meter long terrace dug into the rock. The fossil site was infilled, tent rocks moved and wastes removed or burned.

Reporting for specific materials, systems and methods

We require information from authors about some types of materials, experimental systems and methods used in many studies. Here, indicate whether each material, system or method listed is relevant to your study. If you are not sure if a list item applies to your research, read the appropriate section before selecting a response.

Materials & experimental systems

n/a	Involved in the study
☒	☐ Antibodies
☒	☐ Eukaryotic cell lines
☐	☒ Palaeontology and archaeology
☒	☐ Animals and other organisms
☒	☐ Human research participants
☒	☐ Clinical data
☒	☐ Dual use research of concern

Methods

n/a	Involved in the study
☒	☐ ChIP-seq
☒	☐ Flow cytometry
☒	☐ MRI-based neuroimaging

Palaeontology and Archaeology

Specimen provenance: The specimen was collected in Canada, Nunavut, southern Ellesmere Island, near the eastern arm of Bird Fiord, Nunavut Paleontological Expedition site NV0401, N77°10.235' W86°11.279'. Export Permit via Canadian Museum of Nature (Ottawa) on behalf of Nunavut, August, 2004.

Specimen deposition: To be returned to the Canadian Museum of Nature upon publication, per agreement of Export and Paleontology permits.

Dating methods: No new dates are provided.

☐ Tick this box to confirm that the raw and calibrated dates are available in the paper or in Supplementary Information.

Ethics oversight: No ethics oversight was required by the Canadian Museum of Nature, Nunavut, or the University of Chicago and Academy of Natural Sciences of Drexel University. The field and environmental protocols were approved by the Hamlet of Gris Fiord, Canada; the Iviq Hunter and Trappers of Grise Fiord Canada, and the Polar Continental Shelf Project of Canada.

Note that full information on the approval of the study protocol must also be provided in the manuscript.
