# Supplementary material for: A new elpistostegalian from the Late Devonian of the Canadian Arctic
Source: Nature. 2022 Jul 20;608(7923):563–8. doi: 10.1038/s41586-022-04990-w (PMC9385497; doi:10.1038/s41586-022-04990-w)
Supplement: Supplementary file 6 — A zipped file containing high-resolution images of all figures. [file 41586_2022_4990_MOESM6_ESM.zip › extended_data_1_full_resolution/figure_5.pdf]

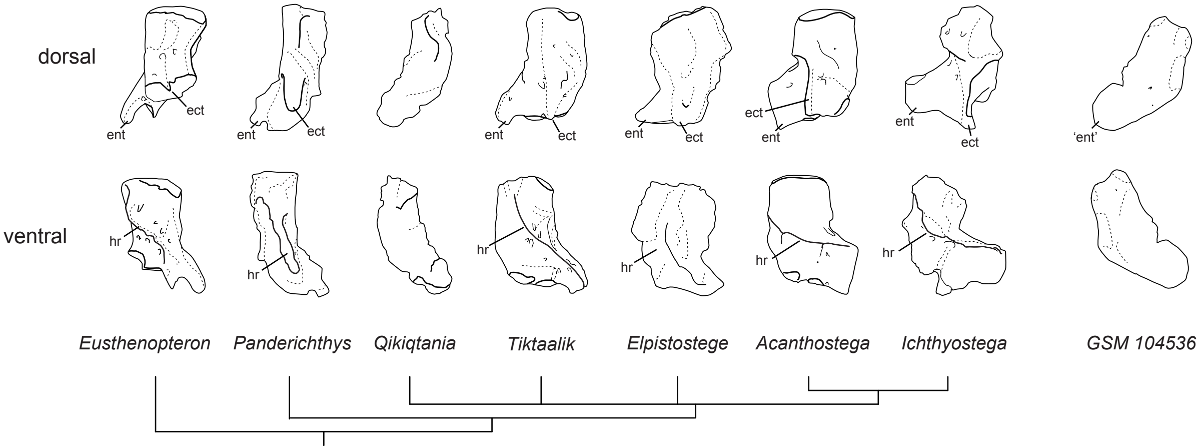
dorsal
ventral
ent
ect
'ent'
hr
Eusthenopteron
Panderichthys
Qikiqtania
Tiktaalik
Elpistostege
Acanthostega
Ichthyostega
GSM 104536
