# Supplementary figures and images for: A new elpistostegalian from the Late Devonian of the Canadian Arctic
Source: Nature. 2022 Jul 20;608(7923):563–8. doi: 10.1038/s41586-022-04990-w (PMC9385497; doi:10.1038/s41586-022-04990-w)

a

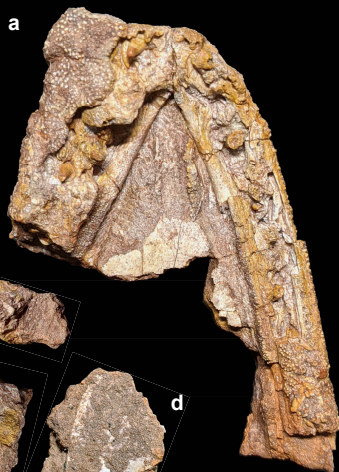

b

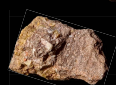

c

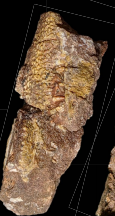

d

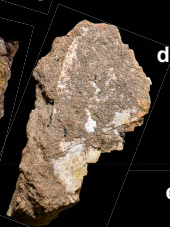

e

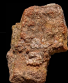

f

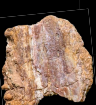

h

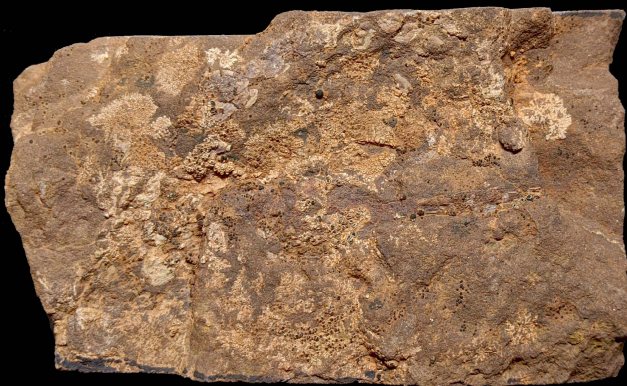

i

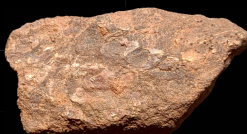

j

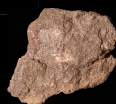

k

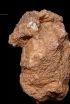

g

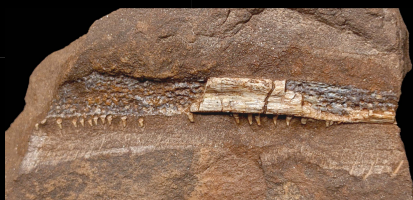

l

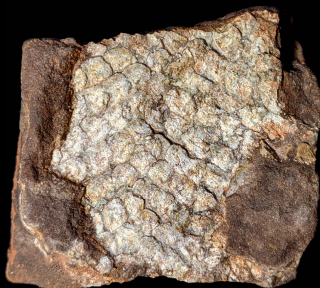

m

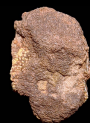

n

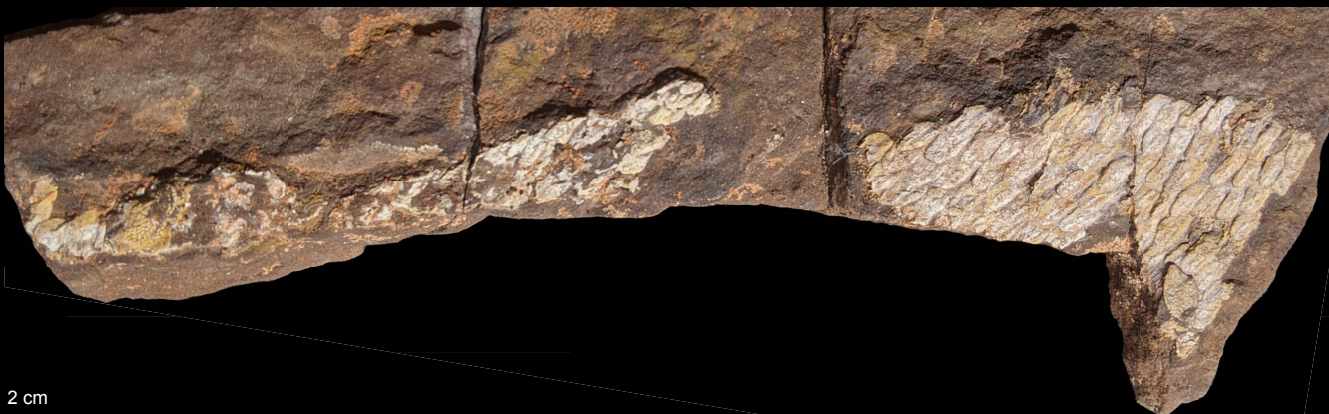

2 cm

Supplement: Supplementary file 6 — A zipped file containing high-resolution images of all figures. [file 41586_2022_4990_MOESM6_ESM.zip › extended_data_1_full_resolution/ExtendedDataFig_2.pdf]

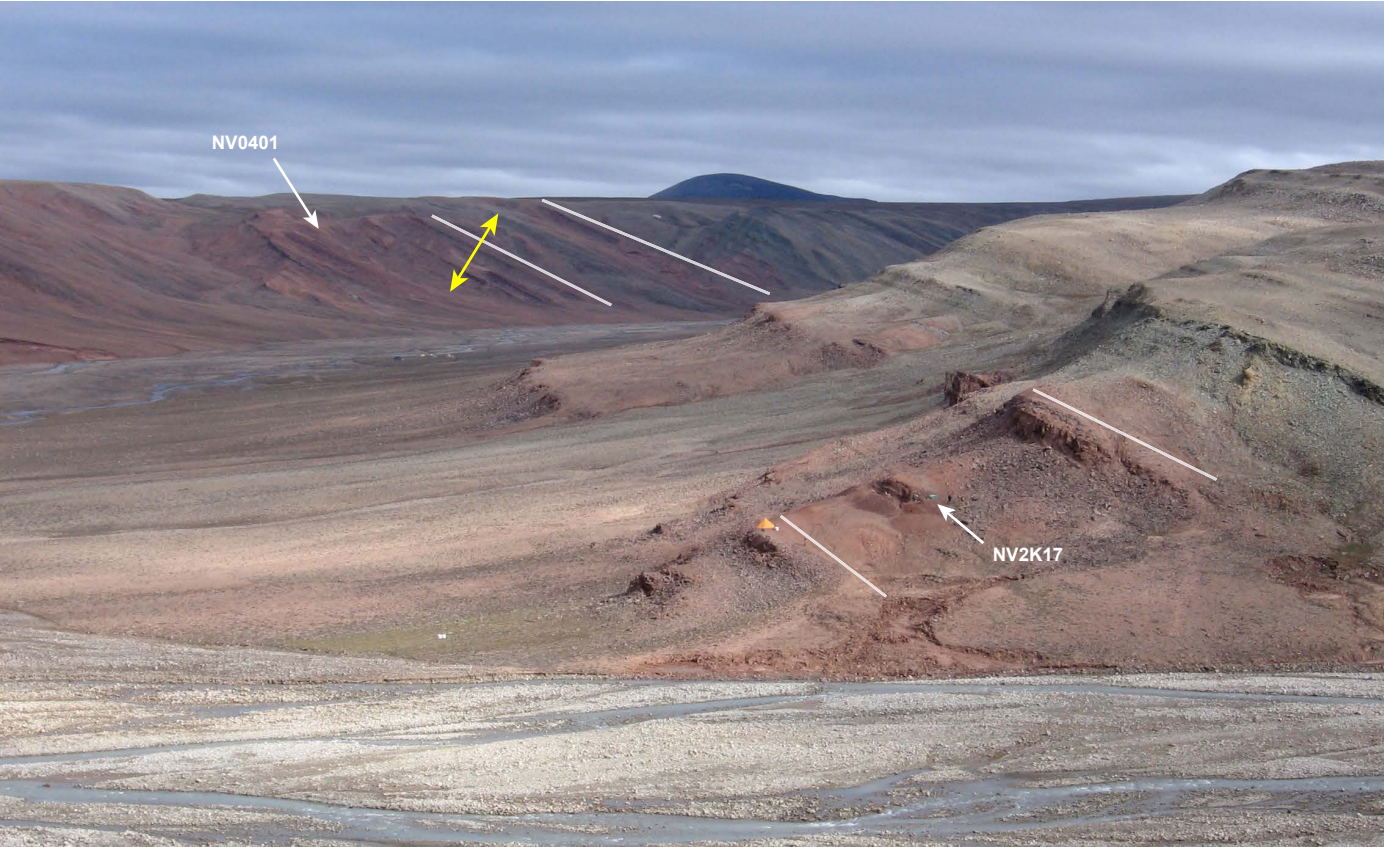

NV0401

NV2K17

Supplement: Supplementary file 6 — A zipped file containing high-resolution images of all figures. [file 41586_2022_4990_MOESM6_ESM.zip › extended_data_1_full_resolution/ExtendedDataFig_1.pdf]

**a**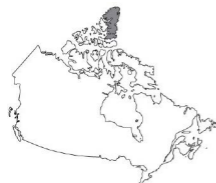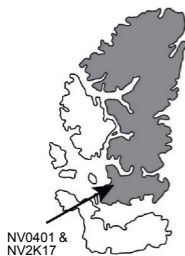**b**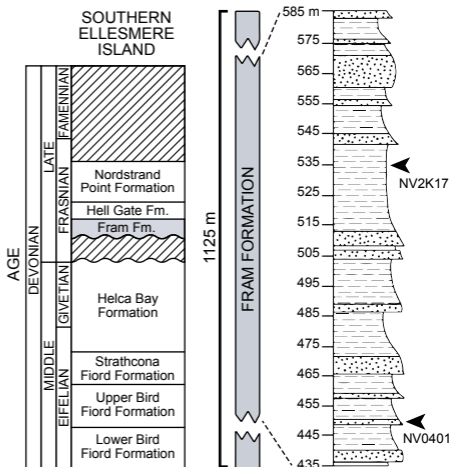**c**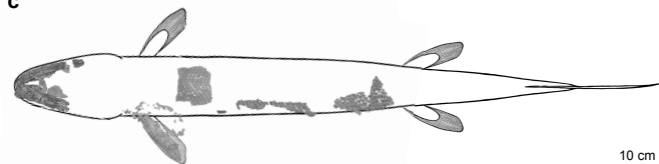

10 cm

Supplement: Supplementary file 6 — A zipped file containing high-resolution images of all figures. [file 41586_2022_4990_MOESM6_ESM.zip › extended_data_1_full_resolution/figure_1.pdf]

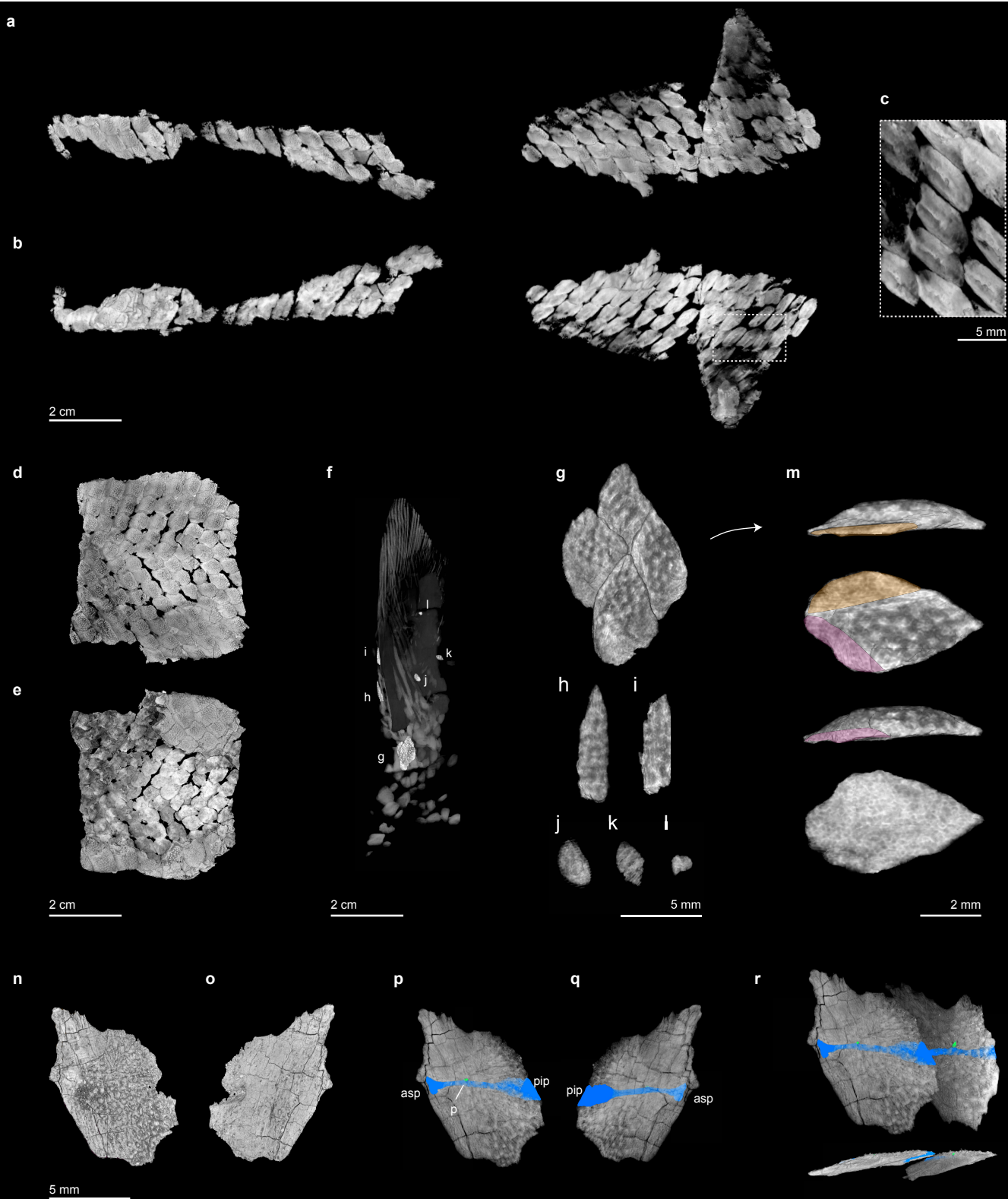

Supplement: Supplementary file 6 — A zipped file containing high-resolution images of all figures. [file 41586_2022_4990_MOESM6_ESM.zip › extended_data_1_full_resolution/ExtendedDataFig_4.pdf]

**a**

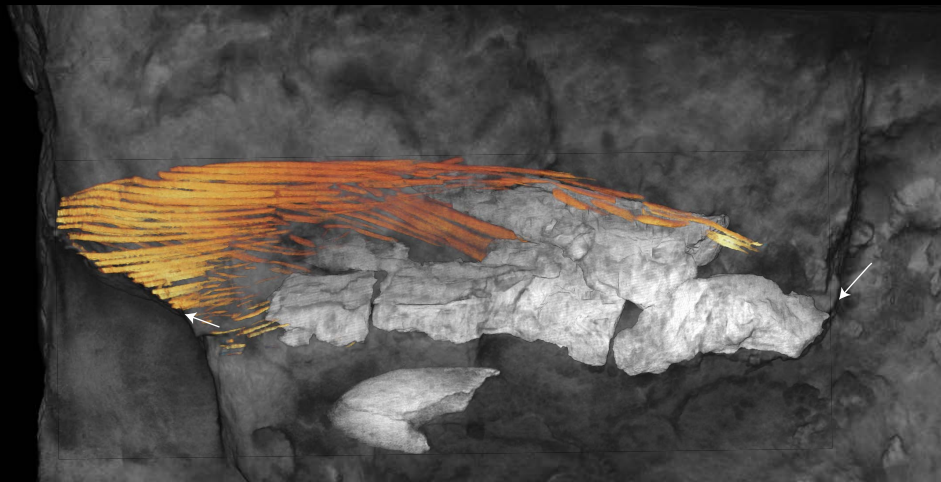

5 mm

**b**

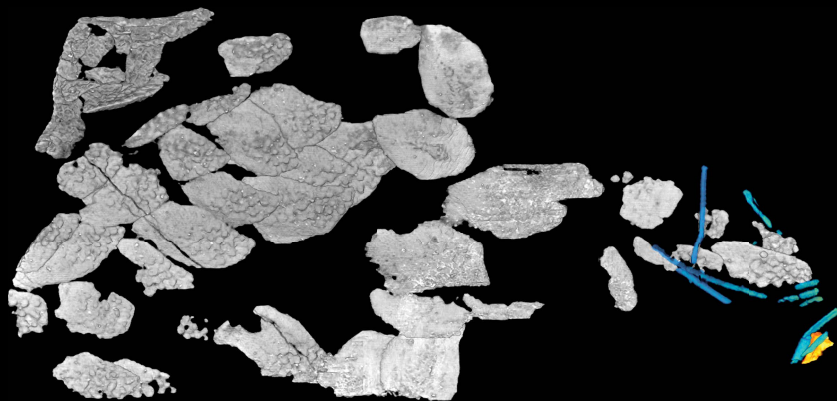

5 mm

**c**

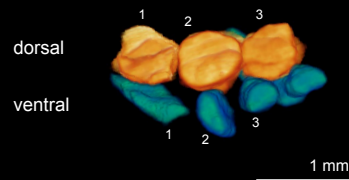

dorsal

ventral

1 mm

**d**

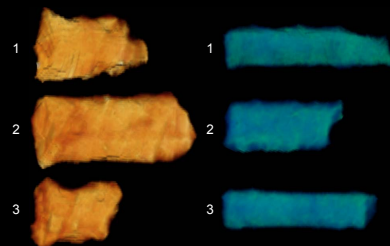

1 mm

Supplement: Supplementary file 6 — A zipped file containing high-resolution images of all figures. [file 41586_2022_4990_MOESM6_ESM.zip › extended_data_1_full_resolution/ExtendedDataFig_3.pdf]

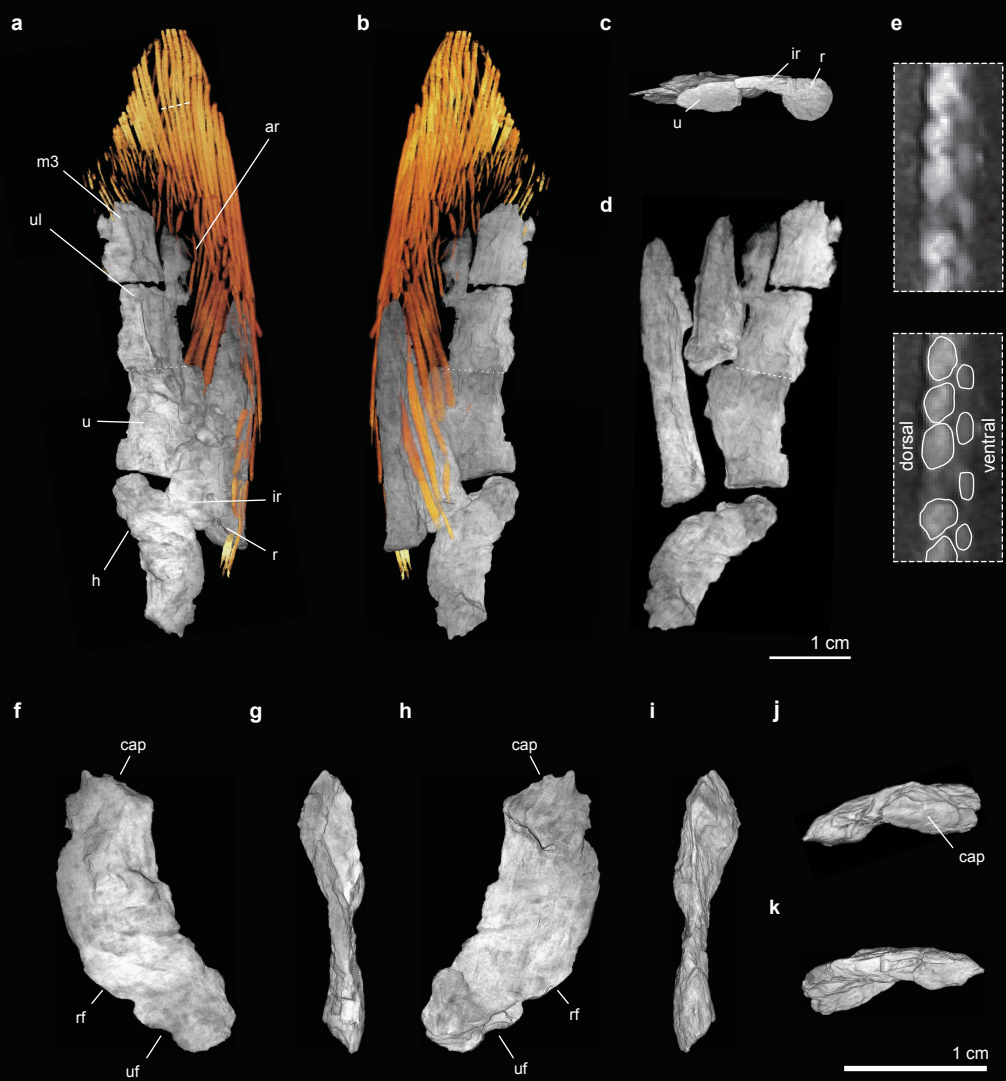

Supplement: Supplementary file 6 — A zipped file containing high-resolution images of all figures. [file 41586_2022_4990_MOESM6_ESM.zip › extended_data_1_full_resolution/figure_3.pdf]

**a**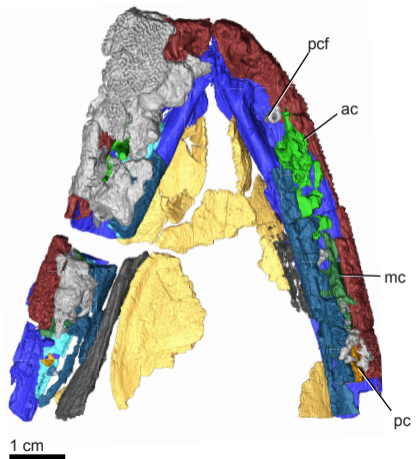**b**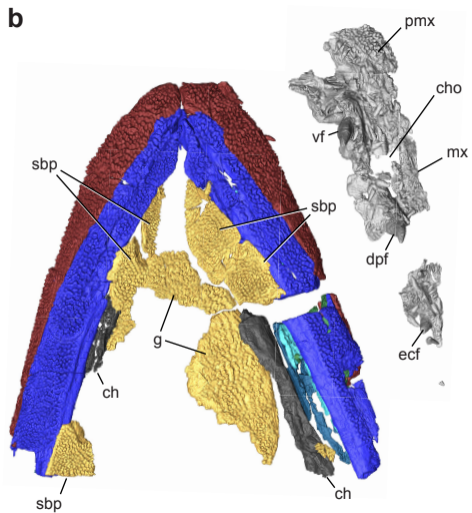**c**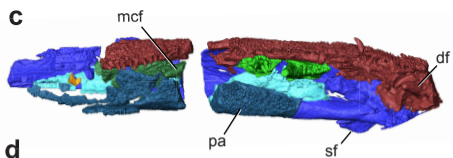**d**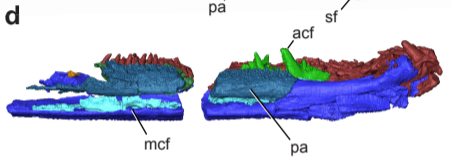**e**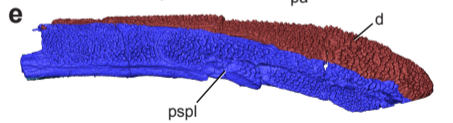**f**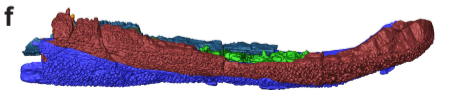

Supplement: Supplementary file 6 — A zipped file containing high-resolution images of all figures. [file 41586_2022_4990_MOESM6_ESM.zip › extended_data_1_full_resolution/figure_2.pdf]
